# ENERGY Pro: Spatially explicit agent-based model on achieving positive energy districts

**DOI:** 10.1016/j.mex.2024.102779

**Published:** 2024-05-28

**Authors:** Erkinai Derkenbaeva, Gert Jan Hofstede, Eveline van Leeuwen, Solmaria Halleck Vega, Juriaan Wolfers

**Affiliations:** a Department of Social Sciences, Wageningen University and Research, Hollandseweg 1, Wageningen 6706 KN, the Netherlands; b Amsterdam Institute for Advanced Metropolitan Solutions, Kattenburgerstraat 5, b. 027W, Amsterdam 1018 JA, the Netherlands; c UARM, North-West University, 11 Hoffman Street, Potchefstroom 2351, South Africa

**Keywords:** ODD+D protocol, Social simulation, Spatial microsimulation, Energy transition, Energy-efficient retrofitting, Amsterdam

## Abstract

This article describes the *ENERGY Pro* agent-based model using the Overview, Design Concept, and Details + Human Decision-making (ODD+D protocol). The model is empirically explicit and aims to investigate the adoption decisions of homeowners in Amsterdam on different energy-efficient retrofitting (EER) measures. Following the ODD+D protocol, this study uncovers the conceptual framework used for model construction, the spatial microsimulation process of expanding the data, and the model implementation details. The article also describes sensitivity analysis, validation results, and how to use and adapt the model. With this article, the authors aim to make the model replicable and accessible to other researchers and inspire them using the combination of social simulation and spatial microsimulation in studying the energy transition.

• The agent-based model is described using the ODD+D protocol.
• The combination of simulation methods is used for constructing an empirical model.
• The model on energy transition can be adapted for other cities.

## Graphical abstract

**Figure:**
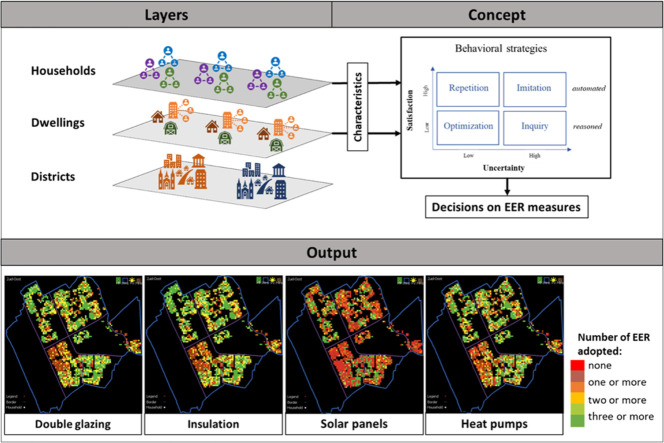

Specifications Table

**Table:** 

Subject area:	Energy
More specific subject area:	Social simulation; spatial microsimulation
Name of your method:	Agent-based modeling; Sensitivity analysis; Population synthesis; Iterative Proportional Fitting; “Truncate, replicate, sample”, Imputation
Name and reference of original method:	Agent-based modeling: Grimm, V., Berger, U., Bastiansen, F., Eliassen, S., Ginot, V., Giske, J., …DeAngelis, D. (2006). A standard protocol for describing individual-based and agent-based models. Ecological Modelling, vol. 198, no. 1–2, pp. 115–126, doi: 10.1016/j.ecolmodel.2006.04.023. https://www.sciencedirect.com/science/article/pii/S0304380006002043
	Müller, B., Bohn, F., Dreßler, G., Groeneveld, J., Klassert, C., Martin, R., …Schwarz, N. (2013). Describing human decisions in agent-based models - ODD+*D*, an extension of the ODD protocol. Environmental Modelling and Software, vol. 48, pp. 37–48, doi: 10.1016/j.envsoft.2013.06.003. https://www.sciencedirect.com/science/article/pii/S1364815213001394 Spatial microsimulation: Lovelace, R., & Dumont, M. (2016). Spatial Microsimulation with R (1st ed.).Chapman and Hall/CRC, doi: 10.1201/9,781,315,381,640. https://www.taylorfrancis.com/books/mono/10.1201/9781315381640/spatial-microsimulation-robin-lovelace-morgane-dumont Lovelace, R., Birkin, M., Ballas, D., & van Leeuwen, E. (2015). Evaluating the Performance of Iterative Proportional Fitting for Spatial Microsimulation: New Tests for an Established Technique. Journal of Artificial Societies and Social Simulation, vol. 18, no. 2, p. 21, doi: 10.18564/jasss.2768. https://www.jasss.org/18/2/21.html Lovelace, R., & Ballas, D. (2013). “Truncate, replicate, sample”: A method for creating integer weights for spatial microsimulation. Computers, Environment and Urban Systems, vol. 41, pp. 1–11, doi: 10.1016/j.compenvurbsys.2013.03.004. https://www.sciencedirect.com/science/article/pii/S0198971513000240
Resource availability:	NetLogo 6.3.0 is available from: https://ccl.northwestern.edu/netlogo/ R (“ipfp”, “mice” packages) is available from: https://www.r-project.org

## Background

In light of the current crises, increasing CO_2_ emissions, climate change, and energy crisis have become environmental commons dilemmas. The commons dilemmas arise when individuals share a common (environmental) resource, e.g., the atmosphere, and their use of that resource negatively affects the welfare of others [2,3]. As energy transition is part of the solution for these dilemmas, sustainable energy behaviors are germane. Sustainable energy behaviors include adopting renewable resources, implementing energy efficiency measures in buildings, and using more sustainable and energy-efficient appliances [4]. The *ENERGY Pro* model aims to investigate such behaviors of households in Amsterdam to understand to what extent households can contribute to tackling these environmental commons dilemmas through achieving Positive Energy Districts (PEDs) [5]. PEDs are energy-efficient, self-sufficient, and carbon-neutral urban areas and are considered one of the possible pathways toward urban energy transition [6,7].

Among different simulation approaches, agent-based modeling (ABM) is the key approach to quantitatively studying the behaviors of heterogeneous agents and their interactions over time [8]. Designed for bottom-up analysis, the ABM helps capture individuals’ emergent behavior and explains more complex macro behavior observed in the real world. The predominant advantage of using the ABM in researching the energy transition is its ability to account for complexity [9]. An energy system is a complex adaptive system comprised of heterogeneous agents and technologies [7]. There are substantive ABM energy transition-related applications, but examples of spatially explicit empirically-driven energy models are still scarce [10].

This simulation model aims to explore how energy consumers become *ENERGY Pro*sumers by adopting different energy-efficient retrofitting (EER) measures. The model is empirically explicit and includes two layers – spatial and social. The spatial layer represents residential buildings in Amsterdam and is informed by the BAG^1^ (Basisregistratie Adressen en Gebouwen) data [11]. The social layer denotes Amsterdam households informed by the WoON (WoonOnderzoek Nederland) Dutch survey 2021 [12] and Census data [13]. The model is developed in NetLogo 6.3.0. Additionally, the model heavily relies on R programming language to perform more complex operations, such as spatial microsimulation and other time-expensive operations.

This article describes the *ENERGY Pro* model [5] using the ODD+D protocol (Overview, Design Concept, and Details + Human Decision-making) following the format of Grimm et al. (2006) [14] and Müller et al. (2013) [15]. The ODD+D protocol is a standardized approach to describe agent-based models and is widely accepted and used as it offers easy-to-read documentation of models and facilitates their replication [[16], [17], [18], [19]]. Following the section on ODD+D protocol, this study also offers sensitivity analysis, model calibration and validation, and describes how to use and adapt the model.

## Method details

This section presents the ODD+D protocol, an extension of the original protocol that includes more information on human decision-making. The protocol consists of three main parts: *Overview, Design concepts*, and *Details*, which are presented within this section.

### Overview

#### Purpose

The purpose of developing the *ENERGY Pro* model is to explore households’ decision-making on adopting EER with a particular focus on double glazing, insulation of walls, roofs, and floors, and the adoption of residential solar photovoltaic (PV) and heat pumps. Using the input survey to mimic the Dutch households, this model aims to understand households’ contribution to urban energy transition in Amsterdam by 2030. A key motivation in developing this model is to allow for simulations of possible policy interventions to inform policymakers on observed energy-related decision-making patterns of homeowners and factors affecting these patterns.

#### Entities, state variables, and scales

The *ENERGY Pro* model includes two entity types – households and EER measures. The household subtypes considered in this study are homeowners and tenants. Though homeowners are the ones who make decisions on adopting EER, tenants are taken into account as they also consume energy and contribute to carbon emissions. State variables of the households and their description are presented in Table 1.

**Table 1:** State variables of households based on WoON 2021.

Variable name	Description	Change
*Socio-demographic characteristics*		
Age	7 age categories: 18–22, 23–26, 27–34, 35–44, 45–54, 55–64, 65+	Dynamic
Ownership	Ownership status of an occupant: owner or tenant	Static
Education	3 categories of highest attained education: low, middle, and high	Static
Household composition	5 categories of household composition: single-person house, couple without children, couple with children, single-parent family, and other	Static
Household size	3 categories of household size: single-person household, two-people household, and three(or more)-people household	Static
Income	Annual disposable household income, continuous variable	Static
Income-cat	5 income categories: less than 21,000, 21,000–30,200, 30,200–42,600, 42,600–59,500, more than 59,500	Static
Wealth	Prosperity indicator that combines income, savings, and debts, continuous variable	Static
Social identity (cohesion)	Indicator of social quality – cohesion ranging between 0 and 1	Static
Contact with neighbors	A household has contact with immediate neighbors – range between 0 and 1 based on the Likert scale with an increment of 0.25, where 0 – totally disagree and 1 – totally agree	Static
Level of life satisfaction	Level of life satisfaction ranging between 0 and 1	Static
House landlord	6 categories of owners of rented houses: housing corporation, municipality, pension fund (or insurance company, investors or broker), private person, family, and other	Static
*Dwelling characteristics*		
Location	Neighborhood (Dutch: wijk) and district where a house is located	Static
Construction year	6 categories: built before 1946, 1946–1980, 1981–1990, 1991–2000, 2001–2010, built after 2010	Static
Dwelling type	2 categories of dwelling type: apartment and non-apartment	Static
Electricity consumption	Annual consumption of electricity in kWh	Dynamic
Gas/heat consumption	Annual consumption of gas/heat in m^3^	Dynamic
Existing insulation	There is insulation (either of roof, walls, floor, or all) in the house, 1 – yes, 0 – no	Dynamic
Existing double glazing	There is double (or triple) glazing in the house, 1 – yes, 0 – no	Dynamic
Existing PV	There is PV in the house or building, 1 – yes, 0 – no	Dynamic
Existing heat pumps	There is a heat pump in the house, 1 – yes, 0 – no	Dynamic
Want to move	Household wants to move in the next 2 years, range between 0 and 1 based on the Likert scale with an increment of 0.25, where 0 – definitely not and 1 – found another home	Dynamic

Households are defined by their socio-demographic and dwelling characteristics. The model is built with a rough assumption that all houses are suitable for adopting the measures. All variables in Table 1 are used to build the model, except for “household composition” and “house landlord”. While “household composition” was used in spatial microsimulation as a constraint variable (see more about this process later in this article), “household size” was used instead in constructing the ABM for computation simplicity. The “house landlord” variable was not used for model initialization but to better understand the context, as the study mainly focused on owner-occupiers’ decision-making.

The categorical variables such as “age”, “income-cat”, “household composition”, and “construction year” in Table 1 are categorized based on the CBS (Statistics Netherlands) classification. This is the standard categorization of these variables in the Netherlands, and it is used in (national) surveys. Some of the survey data were recoded to fit the model's construct. These include the following variables: “contact with neighbors” (scaled based on the Likert scale), “level of life satisfaction” (rescaled), “social identity” (rescaled).

EER measures include building retrofit measures (i.e., double glazing, insulation of roof, walls, floor, and heat pumps) and residential solar photovoltaic. It should be noted that storage batteries (considered an integral part of adopting either PV system or heat pumps, or both) are not common in the Netherlands and, accordingly, are not included in this study. The state variables of these measures are presented in Table 2. Each measure available to households is defined by its price. Each type of technology has a different monetary value, which changes over time. Some measures also have after-lifetime emissions, energy generation, electricity demand, and saved heat. EER technologies are not completely renewable and have minor after-lifetime emissions that should be considered. Electricity generation by PV depends on weather conditions already included in the calculation. Heat pumps have electricity demand to produce heat, while insulation measures save up heat use in houses.

**Table 2:** State variables of EER measures.

Variable name	Description	Change
Type of a measure	Double glazing, insulation, PV, heat pumps	Static
Product price	The monetary value of a product in euros	Dynamic
Price change	Change in product price every year in%	Dynamic
After-lifetime emissions	Some products have minor after-lifetime emissions, tons	Static
Energy generation	Average annual electricity generation by PV is calculated depending on weather conditions in kWh, and average annual heat generation by heat pumps in m^3^	Static
Electricity demand	The electricity demand of heat pumps to produce heat in kWh	Static
Saved heat	Share of heat saved through adopted double glazing and major insulation in m^3^	Dynamic

The *ENERGY Pro* model is spatially explicit. The model's spatial layer includes seven Amsterdam districts with a spatial resolution of individual residential buildings. Each cell or patch in NetLogo represents several buildings; each building and its households have individual characteristics (see Table 1). However, these characteristics are aggregated per cell when the distribution of households or adopted EER measures is loaded (the color gradient is used for visualization purposes). The number of households allocated per cell differs depending on a scale represented on the interface, namely district or city. When only considering cells containing households, there are around 205 households per cell at the city scale. In contrast, at the district scale, there are, on average, 23 households per occupied cell (the example of Zuidoost district).^2^ Therefore, in order to have a higher resolution of the households and obtain more precise information, we focus on one district at a time.

The temporal resolution of the model corresponds to one year (one time step) covering a period of 10 years (2021–2030). The Dutch government aims to reduce carbon emissions by 55 % compared to 1990 levels before 2030 [20]; therefore, 2030 is an important target for transforming energy systems, especially in urban areas that face many challenges. This simulation period is chosen to examine how much households in Amsterdam can contribute to this Climate Agreement goal. A one-year step is chosen because the annual energy balance is the most accepted one for calculating the energy balance of PEDs [7]. Also, EER adoption is a major decision that requires substantial time and investments, therefore, a one-year step works best for modeling this decision. Though a one-year step might limit exploring the emergent behavior of the model, it is a common practice in building models of more complex systems such as the energy system [21].

The main global variables in the model include average households’ annual carbon emissions in Amsterdam and electricity and gas prices changing yearly, and energy price uncertainty, among others. Energy prices change annually based on possible energy market fluctuations, while households’ carbon emissions change according to the decisions of households on the level of energy consumption and adoption of the measures. More information on global variables is offered in Table A.1**.** in Appendix.

#### Process overview and scheduling

At the initialization stage, a setup procedure is executed. First, the spatial layer is set by clearing the entire NetLogo environment and loading the GIS dataset. The GIS procedure resizes the environment based on the coordinate system, allocates buildings to cells, assigns district borders, labels to districts, codes and names to neighborhoods, and buildings’ construction year to cells. Second, the social layer with local and global variables is set by creating a population, assigning the values from the datasets to them, and allocating the households on the map. The setup procedure also creates the legend and, if enabled, a new random seed for reproducibility. Then, the list of similar neighbors is created, and energy generation and emissions are calculated.

**Fig. 1:**
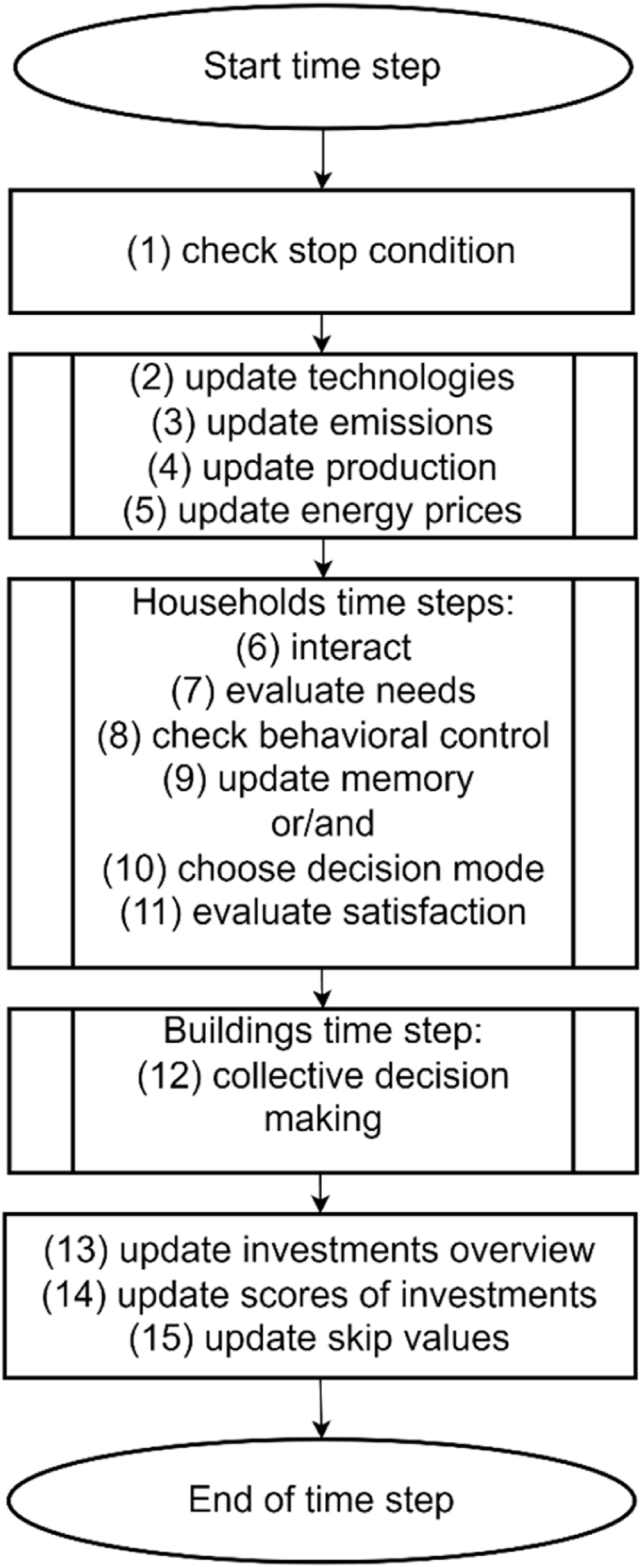
Flowchart of the model's time step.

After the initialization, the *ENERGY Pro* model performs the actions depicted in Fig. 1 each time step. One time step in this model is one year. In sub-step (1), the model checks the number of time steps left. The model stops running when the maximum time steps (i.e., 10) are reached. In sub-steps (2), (3), (4), and (5), the characteristics of the environment are updated. These characteristics include the EER measures’ attributes (e.g., price), carbon emissions, energy generated by households, and energy prices. The environment update is followed by the steps of households. In sub-step (6), households interact with their similar neighbors, after which they evaluate their needs (7) and check if their behavioral control is positive or negative (8). If their behavioral control is positive (meaning they can satisfy their needs), they update their memory (9), and in sub-step (10), they can choose one of the reasoned decisions. If the behavioral control is negative, they skip updating their memory and are left with either automated decision strategies in sub-step (10). In sub-step (11), households evaluate the level of their need satisfaction. Collective decisions on PV adoption in multi-apartment buildings are made in sub-step (12) if activated in the simulation.

Through sub-steps (13), (14), and (15), there are more environment updates. The overview investments procedure updates the investment value of either of the four EER measures adopted by households (13). This procedure is followed by updating the investment score (14). Investment score update is run on a cell basis and assesses how many households living on a cell adopted any measure. They receive a score of 1 for each adopted measure (max. 4). The scores of households living in one cell are aggregated within the interface for visual purposes. In sub-step (15), the characteristics of households that did not participate in a current time step (i.e., did not make decisions) are reset to their default values from the previous time step. These households will start making decisions in the next time step. Important to note that those households that participated in a current time step and are set to skip the following one are not affected by this procedure.

#### Design concepts

The terminology and order of concepts are considered following Grimm et al. (2006) [12] and Müller et al. (2013) [13]. The concepts “Individual sensing”, “Individual prediction”, and “Submodels” are not applicable in this study.

#### Theoretical and empirical background

The *ENERGY Pro* model's design is based on a theoretical framework *Consumat* that was developed by Jager et al. (2000) based on multiple behavioral theories on cognitive processes and underlying driving factors for behavioral change [22]. Energy-related decisions in this model are whether to invest in double glazing, insulation of roof, walls, and floor, and adoption of residential solar panels and heat pumps. The main decision-makers in the model are homeowners. Owners in the Netherlands have the right to make individual decisions on adopting the measures if they live in a single-family house that is a non-apartment dwelling (e.g., detached, semi-detached, terraced, etc.). However, they can only collectively decide about PV adoption if they live in a multi-apartment building sharing a common roof with other residents [23]. According to the statistics, apartment dwellings account for about 85 % of the housing stock in Amsterdam [24], which means most of the PV adoption decisions will be made collectively. Therefore, we differentiate homeowners' individual and collective decisions in this model.

Tenants were also among the adopters based on the WoON Dutch survey 2021 [12], which can be explained by the fact that they might have received the permission of their landlords to adopt the measures. According to Dutch Civil Law [25], they do not have the right to adopt any measure without their landlord's consent. We include the variable on types of landlords of rented houses in the descriptive analysis to observe who is behind the decision-making. We also introduce a scenario where tenants make adoption decisions in order to evaluate their contribution to the energy transition goal.

**Fig. 2:**
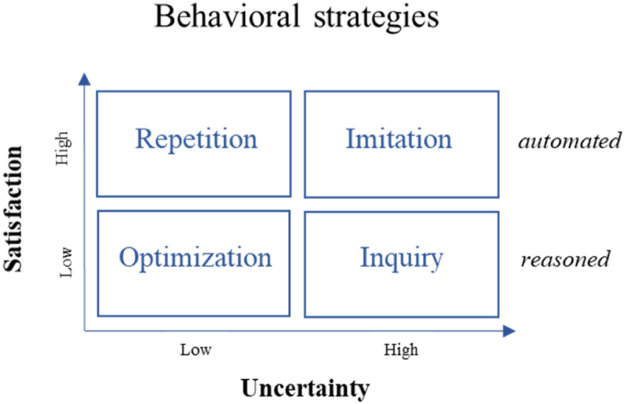
Behavioral strategies based on Consumat [22].

Following the Consumat meta-model, households choose one decision strategy out of four: imitate, optimize, repeat, or inquire. The choice of a strategy depends on their *satisfaction* and *uncertainty* (Fig. 2). Satisfaction and uncertainty are, in reality, subject to social influences that can differ a lot between types of homeowners and types of built areas. If the satisfaction of *consumats* (i.e., agents in Consumat) is high, the consumats will choose one of the automated behavioral strategies and either repeat their previous actions or imitate similar consumats. While the satisfaction of consumats is low, the consumats will choose one of the reasoned behavioral strategies – to optimize their actions by finding a better solution to satisfy their needs or to inquire actions of other consumats (with strong and weak ties) that seem to be satisfying the needs. Uncertainty, in its turn, guides which of those two strategies of automated and reasoned behaviors consumats pursue. Each consumat has a particular level of uncertainty about their decisions and the future in general, as well as uncertainty tolerance that determines to what extent a consumat is risk-seeking. More information on the conceptual framework is uncovered in the *Details* section.

The *ENERGY Pro* model is mainly based on empirical data from the WoON Dutch survey 2021 [12]. The data is available at a household level. The number of households from Amsterdam that participated in the WoON survey in 2021 is limited to 1630, which is insufficient to analyze the city thoroughly. To use the real (even though limited) data and shed new light on available information, we use a spatial microsimulation approach that helps create approximations of individual-level data at high spatial resolution: households allocated to places [26]. A key step in spatial microsimulation is population synthesis, which combines the real individual-level data (with little or no geographical information) from the WoON survey and geographically aggregated data (Census data). As a result of population synthesis in Amsterdam, we created a usable dataset with 447 685^3^ households assigned to a neighborhood based on their characteristics. It is a little smaller than the actual number of households as several neighborhoods have been removed from the synthetic population dataset (see more specifics in the *Details* section).

### Individual decision-making

At each time step, households update their socio-demographic and dwelling characteristics. Before households’ decision-making process starts, they interact with other agents in their social network that are similar. Similar households are chosen based on several aspects discussed in the *Details* section. After interacting with similar agents, households evaluate their needs. Based on the original Consumat framework, there are three categories of needs – existence, social need, and personality [22]. Existence refers to having means for life, such as housing, food, and clothing. Social refers to the agent's place within its network(s), while personality reflects the agent's style and taste (different from others). Within each category, there can be several needs.

This study focuses only on two need categories – existence and social need. We consider the personality need to already be part of the social need as individuals are embedded within social networks, and their preferences are shaped by their interaction with others in their social environment [27]. As such, in the *ENERGY Pro* model, households’ need for existence is their energy need, while their social need is their identity (i.e., belonging to a group, having social status) that can be satisfied if more similar neighbors adopt the same product. More information on constructs of need satisfaction and its calculation are offered in the *Details* section.

To satisfy their needs, households check their behavioral control. Behavioral control is the difference between households’ abilities to consume available opportunities and the resource demand of available opportunities. Opportunities are the products and services (commodities) that an agent can use and have a certain capacity to satisfy the agent's needs (e.g., EER). In this study, abilities include legal rights to adopt EER measures and availability of financial resources, while resource demand includes the availability of EER measures and their prices. More information on behavioral control is in the *Details* section.

Spatial aspects such as house location and proximity between buildings/households impact the energy-related behavior and decisions of households based on technical and social factors [28]. First, house location implies spatial characteristics (location in a particular part of the city) that play a role in decision-making based on technical features such as historical centers or newly built areas imposing possible constraints or creating opportunities. Second, proximity between buildings influences decision-making based on social factors allowing the interaction of (similar) neighbors. Temporal aspects also play a role in the decision process of households. Technology becomes less expensive and more affordable over time, making it accessible to a wider population. Also, the population becomes more experienced and knowledgeable in adopting EER, which will be diffused in the social network through agents’ interaction.

#### Learning/memory

Based on Consumat, memory is a learning tool of agents. Agents learn over time based on their experience and connection to their reference group (i.e., neighbors).^4^ In its memory, the agent keeps track of its adopted measures and knowledge of the agents in its social network. As such, every adopted measure by households or their similar neighbors provides a household with new information and experience. Agents update their memory only in reasoned mode, therefore, when they make their decisions on a behavioral strategy, they choose between optimization and inquiry. More information on memory is offered in the *Details* section.

#### Interaction

In the *ENERGY Pro* model, households interact and create their reference group with others with similar location (neighborhood), age, and income. As such, the households create their social network with their similar neighbors. Each household has a set of similar neighbors and chooses one of them at each time step. However, the chosen neighbor does not necessarily reciprocate (i.e., does not choose back the same household). Similar neighbors might affect households’ decisions on energy consumption behavior and adopting measures if they are satisfied and certain. After interacting and gathering information about their similar neighbors’ experiences, households choose to either imitate their behavior and reduce energy consumption (by max. 25 %) or inquire and adopt one of the EER measures adopted by their neighbors that they still have not adopted.

Households also interact with EER measures by adopting them. This interaction enables households to increase energy efficiency in the house and reduce energy consumption. On the contrary, if these measures are not adopted, households negatively affect the environment by contributing to carbon emissions caused by consuming non-renewable energy and living in an energy-inefficient dwelling. The emissions lead to and are not limited by environmental degradation, health issues, and climate change [29,30].

The interaction of the environment with households also exists. Environment variables (i.e., macro-level variables) such as electricity and gas prices affect households' decision-making on energy consumption and adoption of measures. If energy prices change over time, households make consumption and adoption decisions accordingly. For example, if energy prices increase, households are more likely to reduce their energy consumption as it becomes less affordable and to try investing in EER expecting financial returns on investments.

#### Collectives

Based on the nature of the residential built environment, some collectives in the model, such as multi-apartment buildings, have to make decisions jointly. This applies to the decision to adopt solar panels because such buildings share a common roof. The majority of apartment owners’ association (VvE) members must agree on this decision (often, a two-thirds majority is sufficient) before it can be implemented [23,31].

#### Heterogeneity

The households in the model are heterogeneous. They differ in terms of their socio-demographic and dwelling characteristics. The survey data for developing this empirical model are based on a stratified sampling method to represent Amsterdam's population groups. Therefore, exchanging one homeowner with another would affect the simulation. In their decision-making, the homeowners generally differ in their satisfaction and uncertainty and, therefore, their chosen behavioral strategies.

#### Stochasticity

Households perform actions ordered by the modeler consecutively in random order. This applies to actions related to households’ decisions, interaction with their similar neighbors (they randomly choose a different similar neighbor every time step from the list created during the setup procedure), decisions’ implementation, etc. This is the standard mechanism of NetLogo.

#### Observation

**Fig. 3:**
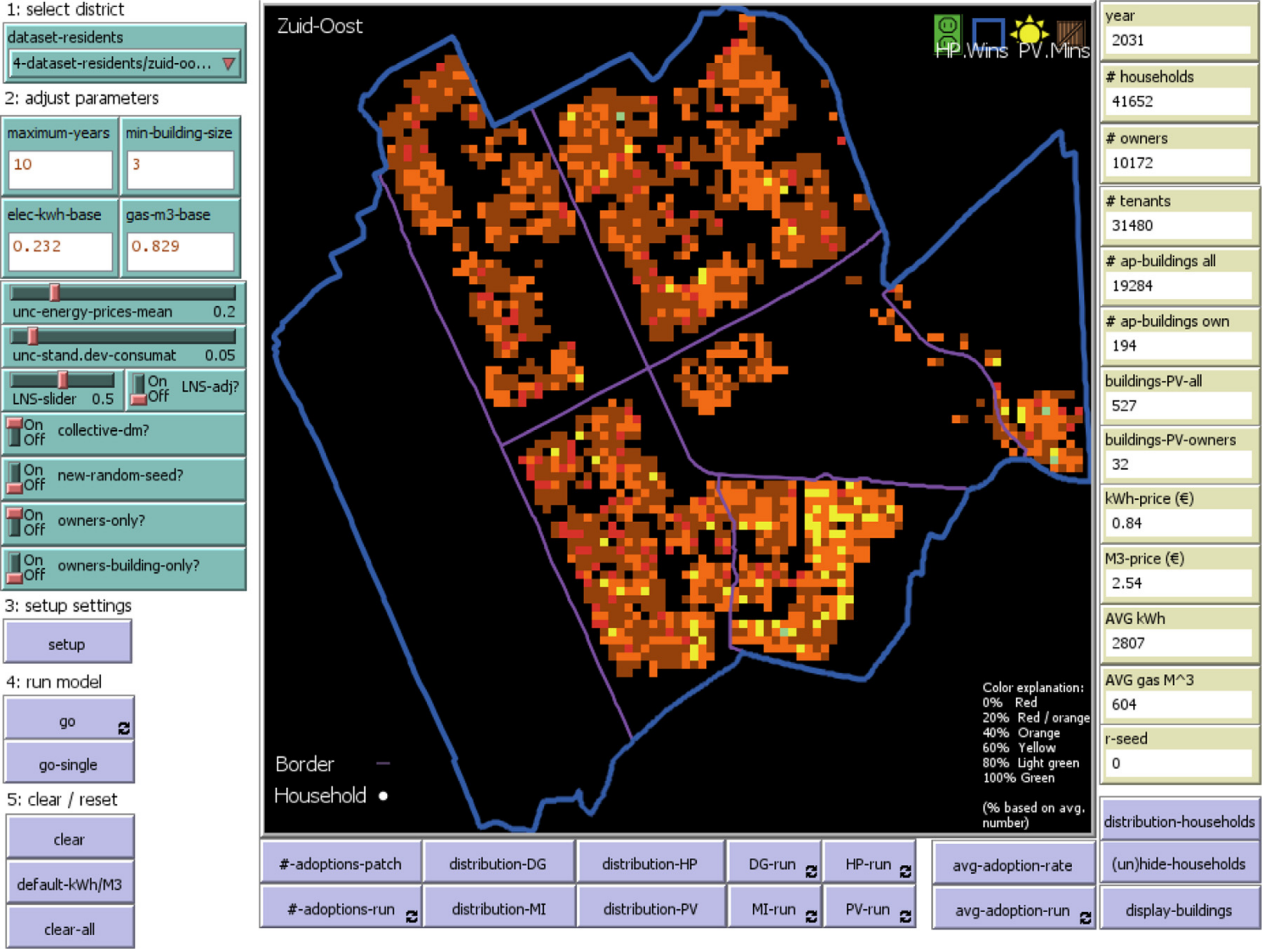
Energy Pro model interface (example of Zuidoost) [1].

As this study examines the EER adoption decisions of homeowners in Amsterdam, the graphical model output can demonstrate the provisional average adoption rate of all EER measures between 2021 and 2030 for all the city districts. Fig. 3 presents an example of the uptake of EER measures in Zuidoost over the simulation period. Based on the run shown in Fig. 3, the average EER adoption rate is higher in the southwestern neighborhood of “Gein” of Zuidoost. Yellow cells mean that the households that occupy them adopted between two and three different types of EER on average. In contrast, red cells spread across the district show that those households occupying the cells did not adopt any measures. We generally observe more orange and brown cells showing that the average number of adopted EER types varies between one and two. There is a limited number of cells that adopted between three and four EER types colored light green.

**Fig. 4:**
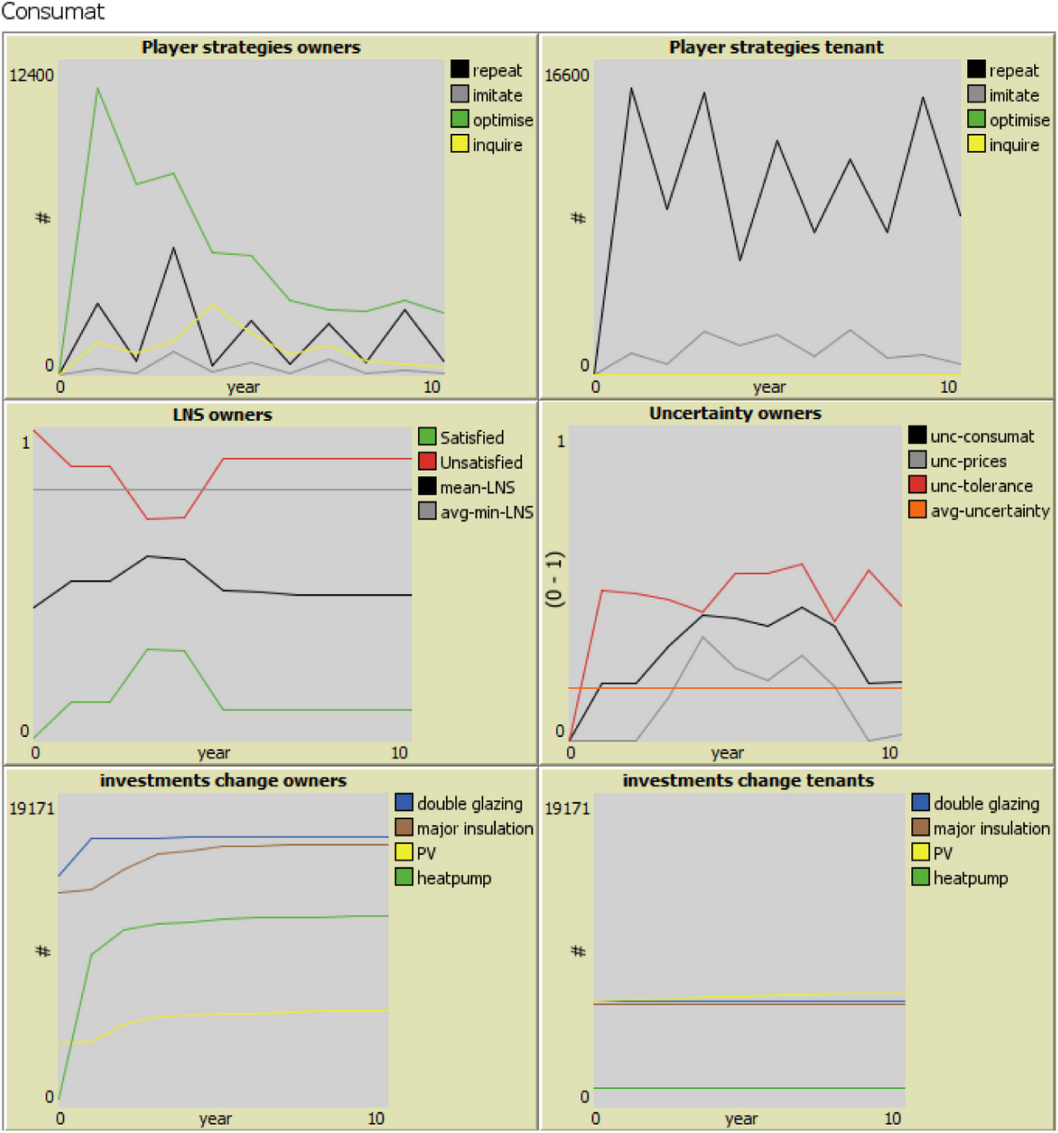
Consumat-related parameters (example of Zuidoost) [1].

The model's interface also includes plots on Consumat-related parameters (Fig. 4). The upper two plots on players’ strategies show the four decision strategies households choose every time step. The middle two plots demonstrate the homeowners’ level of need satisfaction (LNS) and uncertainty based on which they decide on a strategy. We observe that in Zuidoost, homeowners mostly optimize at the beginning of the simulation because of their low LNS and uncertainty, especially during the first time steps. The largest number of homeowners optimizing was in time step 1 (the year 2022). In the next time steps, the number of homeowners optimizing has been decreasing due to the larger number of homeowners with a higher LNS, but also growing uncertainty. The smallest number of homeowners chose imitating due to their higher LNS and higher uncertainty. We also observe sharp kinks in these plots as the data collected per time step, and the model has a coarse time granularity. The last two plots in Fig. 4 show the cumulative number of EER measures adopted over time. When the option of including tenants in decision-making is switched on, the model calculates their EER uptake. Under the baseline scenario, when only homeowners make adoption decisions, we can observe that in Zuidoost, the most adopted measures are double glazing and insulation, while the least is PV. We can also observe a rapid uptake of heat pumps.

#### Details

In addition to this section's standard subsections based on the ODD+D protocol, we also elaborate on the Consumat framework, synthetic population, and data imputation.

### Implementation details

The model is developed in NetLogo 6.3.0. At initialization, the model reads data from three files. First, BAG data includes information on local registered addresses and buildings, including their construction year. Second, the synthetic population dataset is based on WoON Dutch survey 2021 and Census data. While the BAG dataset is used for setting up the spatial layer, the synthetic population is used for setting up the social layer. Both the BAG data and the WoON data can be accessed by researchers upon request from their sources. Finally, the third dataset contains characteristics of EER measures based on openly available data on the internet.

#### Consumat

The basis of each Consumat behavioral strategy is determined by the level of satisfaction and uncertainty of decision-makers, which are in turn influenced by their individual characteristics. A decision-maker chooses the *Repetition* when their level of satisfaction exceeds the accepted minimal level and their uncertainty level is below their threshold of uncertainty tolerance. This indicates that the decision-maker is highly satisfied and certain, and there is no need to change their behavior. Repetition of satisfactory behavior is a central mechanism behind the development of habitual behavior [32].

When a decision-maker's level of satisfaction exceeds the accepted minimal level, but their uncertainty level is higher than their threshold of uncertainty tolerance, they will choose the *Imitation* strategy. In this scenario, the decision-maker is still satisfied but highly uncertain, which leads them to consider behaviors performed by peers whom they trust and care about (strong links) and imitate them. This behavioral strategy is driven by the social need to be part of a particular society or group, and successful behaviors performed by peers are likely to influence decision-makers to copy them when they are uncertain.

However, when a decision-maker's level of satisfaction falls below the accepted minimal level, and their uncertainty level exceeds their threshold of uncertainty tolerance, they will choose the *Inquiry* strategy. This indicates that the decision-maker is unsatisfied and uncertain and needs to find a better solution to meet their needs. They will seek interesting opportunities used by peers who are not necessarily close (weak links).

On the other hand, when a decision-maker's level of satisfaction falls below the accepted minimal level, and their uncertainty level is below their threshold of uncertainty tolerance, they will choose the *Optimization* strategy. This behavioral strategy is chosen by those who are unsatisfied but quite certain and, therefore, are open to any available opportunity and all possible behavioral options.

**Fig. 5:**
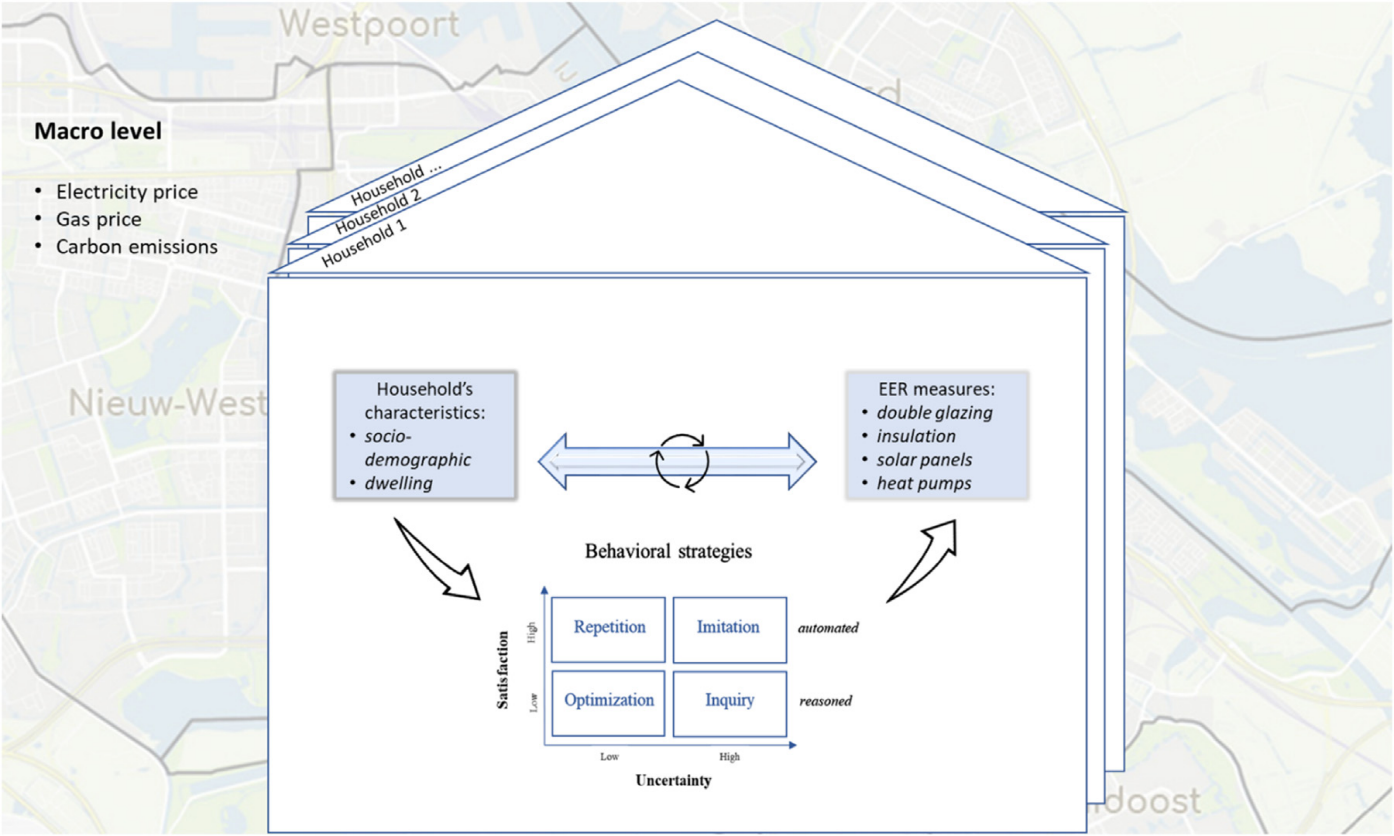
A conceptual overview of the decision-making process in the *ENERGY Pro* model.

The conceptual framework of the *ENERGY Pro* model incorporates micro and macro-driven factors (Fig. 5). Micro-level factors are represented by individual characteristics (socio-demographic and dwelling) of households that affect their satisfaction and uncertainty levels and, ultimately, the choice of a Consumat behavioral strategy. Behavioral options include different ways of adopting or not adopting EER. Adoption or non-adoption decisions impact households’ characteristics as well as households’ characteristics affect their decisions. It creates a feedback loop in the system. In turn, macro-level factors are represented by some global variables that influence all households but to a different extent. Also, households’ decisions affect macro-level factors by contributing, on average, e.g., to increasing or reducing carbon emissions.

#### Consumat parameters

The model calculates several parameters based on the Consumat, including the level of need satisfaction and behavioral control (BC).

#### Level of need satisfaction

In accordance with the Consumat meta-model, agents evaluate their need satisfaction, which is a product of all need categories and ranges between 0 and 1. Weights of need categories depend on agents’ values for each (some need categories might be more important than others, and it differs for each consumat). This study considers the need categories equally important for all households and assigns a similar weighting factor for both needs. This assumption is made to simplify the model and due to a lack of data. The level of need satisfaction for this model is calculated based on the following formula that is adapted for this study from the Consumat [33]:

$$LNS=LNSe^{0.5}\times LNSs^{0.5}$$

*LNS* refers to the level of need satisfaction. A household is satisfied when its LNS is above the minimum level defined by LN*S_min_* (the minimal level of need satisfaction that differs across all households). In this study, the proxy for LN*S_min_* is the level of life satisfaction (Table 1). LN*S_e_* and LN*S_s_* are the level of existence need satisfaction and the level of social need satisfaction, respectively. The level of existence need satisfaction represents the ability of a household to meet its annual energy demand (Table 1), taking into account its disposable annual income (Table 1), and is calculated as the following:

$$LNSe^{0.5}={\left(income-energy\;costs\right)}^{0.5}$$

where

energycosts=elect.cons×elect.price+heatcons×gasprice

As LNS ranges between 0 and 1, the difference between income and energy costs must be rescaled before exponentiation. For this purpose, we use the *income-cat* variable (Table 1) and assign its categories to a number on a scale between 0 and 1 (more precisely, assign “less than 21,000″, “21,000–30,200″, “30,200–42,600″, “42,600–59,500″, and “more than 59,500″ to 0, 0.25, 0.5, 0.75, and 1, respectively). Then, we allocate the difference between income and energy costs to one of the values in the abovementioned scale accordingly. After that, the value can finally be exponentiated to the power of 0.5.

While the income variable remains constant throughout the simulation period, the consumption of electricity and gas/heat will change every time step for different reasons (e.g., previously adopted measures or increasing energy prices can cause energy demand reduction). Electricity and gas prices are global variables; while the prices are known for the first two years of the simulation period, they fluctuate over the rest of the time due to uncertainty. As such, the prices are known and set for 2021 and 2022. In 2023, the government introduced energy price caps (maximum tariffs) for the following usage ceilings: 0.40 euros per kWh up to 2900 kWh of electricity used and 1.45 euros per m^3^ up to 1200 m^3^ of natural gas used [34]. The price cap scheme was introduced to help households with soaring energy prices. During this time step, the model determines whether these price caps are relevant. If households exceed the energy consumption ceilings (electricity or gas, or both), the energy amount within these ceilings will be charged according to the price caps, while the excess will be charged with the energy price accounting for price uncertainty.

The energy price uncertainty ranges between 0 and 1 and is normally distributed with a default mean of 0.2 with a standard deviation of 0.1. The price uncertainty can be determined at the beginning of the run time and can be increased or decreased. The price uncertainty with a mean up to 0.4 will have a standard deviation of 0.1, while with a mean above 0.4, the standard deviation will increase to 0.2, and finally, with a mean of 1, it will increase to 0.4. This condition is introduced because, with the higher uncertainty, there is a higher increase in energy price. All these values are taken arbitrarily, as it is unknown how energy prices will change in reality.

After the price uncertainty is determined, the energy price for each following time step (starting from time step 2) is calculated. As such, the electricity price in every new time step is a value from the previous time step taken as a mean with a standard deviation calculated as a fraction of price uncertainty and the previous value of the electricity price. The gas price in every new step is calculated similarly but with a higher standard deviation (a fraction of price uncertainty multiplied by 4 and the previous value of the gas price) because gas naturally has a higher price than electricity and, thus, will increase by a higher fraction.

The level of social need satisfaction represents the relationship between a household's social identity or cohesion (Table 1) and the adoption of the same product by a similar neighbor – *same-product* (the condition is checked in the model). The *social identity* variable is divided by 10 before the initialization, so it ranges between 0 and 1, saving the model's running time. The *same-product* variable is assigned a value of either 0.5 (if the condition does not hold) or 1 (if the condition holds). The social need satisfaction is calculated as the following:

$$LNSs^{0.5}={\left(social\;identity\times same-product\right)}^{0.5}$$

LNS plays a central role in determining behavioral strategies that households want to follow. When households are satisfied, they will engage in automatic processing (repetition or imitation) and skip the next time step. In contrast, dissatisfied households engage in reasoned processing (optimization or inquiry) and start the new round following all Consumat steps.

#### Uncertainty

The choice of households on behavioral strategy from automatic or reasoned processing will depend on their *uncertainty level*. In the *ENERGY Pro* model, an agent's uncertainty is represented by the probability of moving out (Table 1), which is a proxy ranging between 0 and 1 with an increment of 0.25. As this probability might change within a decade, we added some noise with normal distribution and a standard deviation of 0.05 to this value (this value is taken arbitrarily). Also, the uncertainty of agents depends on energy price uncertainty that is global and similar for each household. As such, households’ uncertainty is calculated in the model as a sum of their personal uncertainty and energy price uncertainty:

$$U=agent^{\prime }s\;uncertainty+energy\;price\;uncertainty$$

A household is uncertain when its uncertainty level is above the uncertainty tolerance. Uncertainty tolerance is normally distributed with a mean of 0.5 with a standard deviation of 0.1. The mean is taken as 0.5 as the Netherlands scores 53 (in the range between 0 and 100, where 100 is large) at the Uncertainty Avoidance dimension of culture theory by Hofstede [35].

#### Behavioral control

Behavioral control is a difference between abilities and resource demand. The primary ability to adopt EER includes the legal rights to do so (only owners can adopt). Another ability is the availability of financial resources – wealth (Table 1), while resource demand includes the availability of EER products (which we assume are available in the market) and their prices (Table 2). After checking the ownership of a household, behavioral control is calculated as the difference between financial ability and a price for a measure that has not been adopted yet as the following:

BC=wealth−productprice

Households’ abilities influence what behavioral options are available and largely determine whether they can satisfy the needs. The fact that households can adopt the measures only when they have sufficient funds and legal rights prevents them from continuously investing in adopting EER whenever they remain unsatisfied. If the behavioral control of a household is smaller than 0, the consumption of an opportunity is impossible. The higher the BC, the easier consumption of the opportunity becomes. In addition, if a household's energy bills represent a high percentage of its income (more than 10 %),^5^ it cannot afford to invest in EER. Thus, households encountering this condition make only a “repeat” decision. If the behavioral control is positive, households update their memory. In its memory, the agent keeps track of its knowledge and experience of adopted measures and the information of the agents in its social network. Agents update their memory only in reasoned mode, therefore, when they make their decisions on a behavioral strategy, they choose between optimization and inquiry. Accordingly, if the behavioral control is negative, agents choose a strategy of the automated mode – repetition or imitation.

#### Memory

In this model, memory value starts from 0.1 as all households are assumed to hear and know the general information from media about climate change, energy crisis, energy transition, etc. In addition, the households have their own beliefs about it; however, there is a lack of data with regard to this factor. Thus, to incorporate the initial beliefs of households, the value in the range of 0 and 1 with an increment of 0.1 is assigned to each household randomly. The memory also increases by 0.2 in two cases: either if a household adopted any measure already and presumably has information and experience in it or when a household interacts with a similar neighbor and receives information about the neighbor's experience. In the latter case, the impact of interaction (having contact with a similar neighbor) should be at least 0.5, which means there is an information exchange. Finally, when it reaches 1, the memory resets to the sum of the initial value of 0.1 and the initial belief value (different for each household). In the model, this reset function is called “forget-old-information” and is used to reduce the impact of old information. Households do not adopt if their memory is lower than 0.5, meaning they do not have enough information.

The similarity of households is based on location, age, and income (Table 1). Location is a neighborhood (Dutch: wijk) where a household resides. There are 99 neighborhoods in the city of Amsterdam (5 neighborhoods out of which are dropped based on the analysis of their representativeness). Households also check their similarity based on the age category. In this study, age refers to the age of a representative of a household who undertook the survey (overall, there are 7 age categories). The last aspect that is checked for the similarity between households is income. In this case, we use the income-cat variable with 5 categories, and if households fall under the same household income category, they are considered similar.

#### Synthetic population

**Fig. 6:**
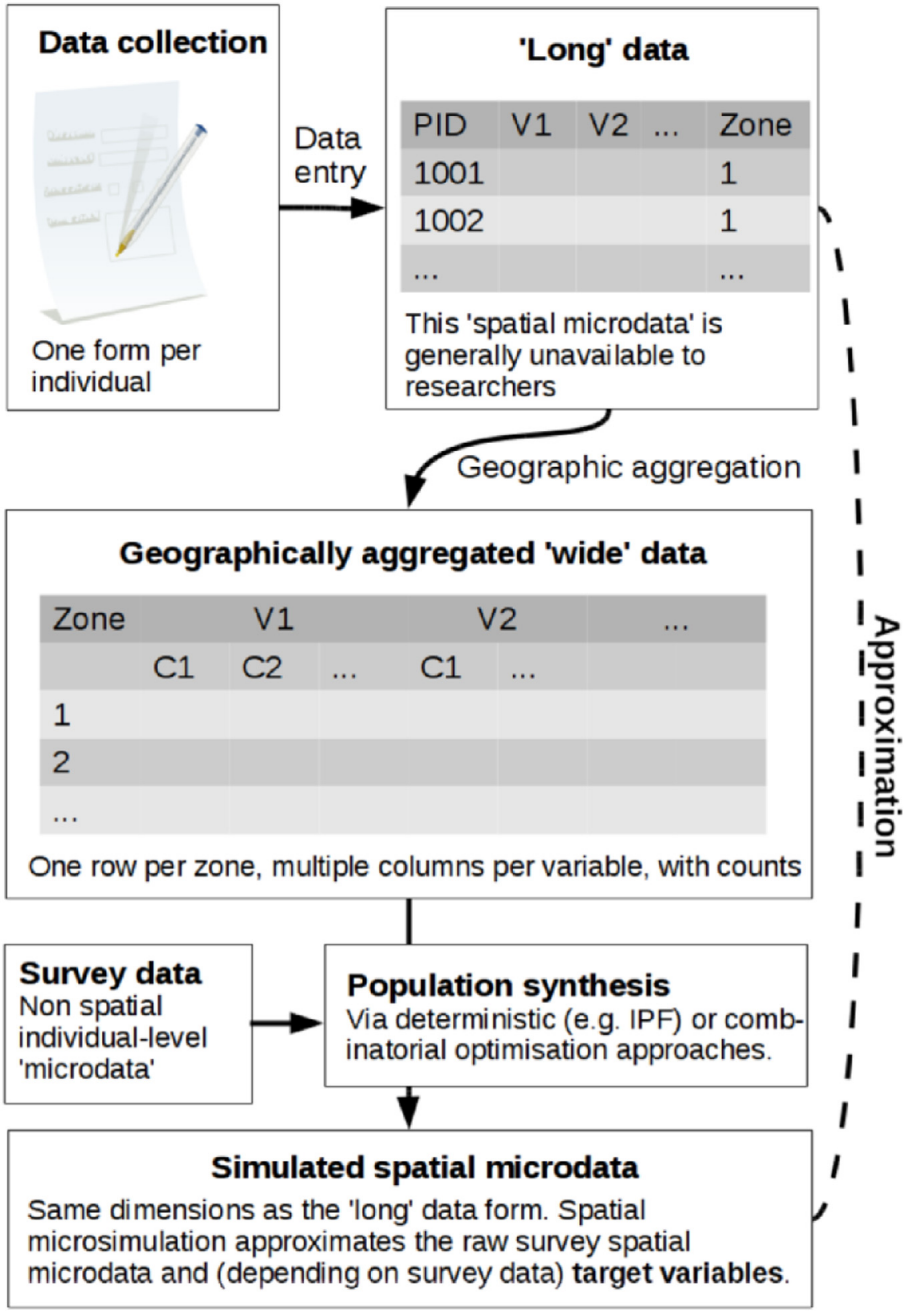
Schematic of population synthesis [38].

The synthetic population was created using the spatial microsimulation method and R package *ipfp,* known for its computation speed and simplicity [36]. Spatial microsimulation involves sampling rows (observations) of survey data to generate lists of individuals for geographic zones that expand the survey to the population of each geographic zone considered [37]. While most publicly available census datasets are aggregated, and individual-level survey data with geographical details are unavailable for confidentiality reasons, this method overcomes the challenge by combining census and survey data to simulate geographically specific populations (Fig. 6).

In this study, we use the Iterative proportional fitting (IPF) technique, which is one of the key techniques in spatial microsimulation [37]. This technique enables the calculation of the maximum likelihood of the presence of given individuals from survey data in specific zones based on census data. In other words, the method allocates all households from the sample survey to each small area and then reweights each household for each small area. The IPF algorithm is implemented using the following formula [36]:

$$w_{i,z,t+1}=w_{i,z,t}*\;\frac{cons_{z,v,ind_{i,v}}}{\sum_{j\in I}w_{j,z,t+1}*I\left(ind_{j,v}=\;ind_{i,v}\right)}$$

In this formula, *I* represents the set of households, *Z* – the set of zones (i.e., neighborhoods), *V* – the set of variables, and *C_v_* – the set of categories for variable *v ∈ V* (e.g., income categories).

The matrix *ind* is a two-dimensional array (based on survey data) where each row *i ∈ I* represents a household and each column *v ∈ V* a variable. As such, the value of the cell *ind_i,v_* is the category of the household *i* for the variable *v*. Then, a three-dimensional array *cons* (based on census data) represents constraining count data: *cons_z,v,c_* is the number of households corresponding to the marginal for the neighborhood *z ∈ Z (*e.g., neighborhood Gein*)*, in the variable *v ∈ V* (e.g., household income) for the category *c ∈ C_v_* (e.g., income category “30,200–42,600″). Given the example, this means that *cons_z,v,c_* denotes the total number of households in the neighborhood Gein with annual income ranging between 30,200–42,600 euros. In this study, constraint variables are household income, household compositions, dwellings’ construction year, and living area size. These constraint variables are selected following the previous studies’ findings on factors affecting the adoption of EER measures [[39], [40], [41], [42]].

*I(ind_j,v_ = ind_i,v_)* is the indicator function that checks the condition whether the category of the variable *v* for the household *j* (i.e., any household in the set *I*) is the same as the category of the variable *v* for the household *i* (i.e., specific household in the set *I* being considered in the current iteration). The indicator function outputs 1 if the condition is true and 0 otherwise. This is a process of selecting only those households *j* that share the same category as the household *i* for the given variable *v*. The sum in the denominator is over all households *j* in the set *I*. As *ind_i,v_* is the category of the household *i* for the variable *v*, the denominator corresponds to the sum of the actual weights of all households having the same category in this variable as *i*. The weights are redistributed such that the data follows the constraint concerning this variable.

Finally, the weight matrix *w(i,z,t)* determines how representative each household is of each neighborhood with *i* corresponding to the weight of the household in the neighborhood *z* during the step *t* (i.e., iterations over constraints). However, the IPF generates fractional weights making it difficult to use the results as a final table of agents needed as input for an ABM. Therefore, converting the fractional weights into integers with a minimum loss of information is important before we use it in the *ENERGY Pro*-model.

The “Truncate, Replicate, Sample” (TRS) integerization method is one of the probabilistic methods that has proven to be more accurate than other methods (i.e., deterministic) [37]. This method constrains the maximum and minimum integer weight resulting from integers just above and under each fractional weight based on probability [38]. The TRS consists of three steps: (1) *truncate* all weights by keeping only the integer part, (2) *replicate* agents by considering these integers as the number of each type of agent in the zone, (3) *sample* according to the probability of an agent to appear in the zone.

The next step after integerization is validation. The validation of the created synthetic population included the following goodness-of-fit measures: fit between constraints and estimates based on a correlation coefficient (also for each neighborhood), distribution of households based on their household size categories per district, comparing the number of districts and neighborhoods created with the census data, and standardized absolute error (i.e., relative error). We conduct several evaluation measures to ensure the validity of the dataset created.

To evaluate the fit between constraints and estimates and their correlation for each neighborhood, we used Pearson's coefficient *r* as it is the most commonly used measure of aggregate level model fit for internal validation [36]:

$$r=\;\frac{\frac{1}{n}\;\sum_{i=1}^{n}x_{i}y_{i}-\;xy}{\sqrt{\frac{1}{n}\sum_{i=1}^{n}x_{i}^{2}-x^{2}}\;\sqrt{\frac{1}{n}\sum_{i=1}^{n}y_{i}^{2}-y^{2}}}$$

This formula corresponds to the covariance divided by the product of the standard deviation of each vector *x* and *y* (observed and estimated). If both vectors have the same values and the covariance is equal to the product of the standard deviation, the *r* coefficient is then close to 1 and the fit is perfect. This measure is sensitive to outliers in the vectors, which means if only one category has a bad fit, the *r* value is very affected, and therefore, should be reliable.

Another evaluation we undertook to validate the dataset is measuring standardized absolute error, also called relative error *RE* [36]:

$$RE=\frac{TAE}{P*n\_var}=\;\frac{\sum_{ij}\left|obs_{zc}-\;est_{zc}\right|}{P*n\_var}$$

The RE is the proportion of the total absolute error *TAE* to the product of the total population *P* and the number of variables *n_var. TAE* is the sum of errors based on observed *obs* and estimated *est* values for each constraint category *c* and each neighborhood *z*.

Based on these tests, we omitted five neighborhoods (out of a total of 99) and one district (Westpoort) from the analysis that did not pass the validation due to a lack of data in the survey. The details concerning the goodness-of-fit measures and their outputs are offered in Table B.1. and Table B.2**.** in Appendix.

#### Data imputation

After creating the synthetic population, there are still some missing values for three variables: electricity consumption (12 % missing), gas consumption (12 % missing), and landlord (8 % missing). We impute the data to avoid omitting the observations with unknown values and biases caused by them. Data imputation is a process of replacing missing data with an estimated value based on other available information. Imputing the data with about 10 % missing values is acceptable. To impute the data, we use R package mice. After all missing values have been imputed, the data can be treated and analyzed following standard approaches for complete data.

#### Initialization

The initial state of the model is determined by the input files, which include geospatial data on residential buildings, household characteristics, and information on various EER measures. The number of households and dwellings remains the same in all runs; their allocation to buildings is based on location, dwelling type, and construction year. Due to the lack of precise household coordinates, households may be assigned to different buildings within a neighborhood each time the model runs, depending on the random seed. Each cell is shared by several buildings. Additionally, the model sets initial electricity and gas prices, as well as carbon emissions, for the year 2021.

### Input data

After initialization, this model does not input further external data.

## Method validation

This section presents sensitivity experiments, model calibration and validation, and how to use and adapt the model.

### Sensitivity experiments

#### OFAT sensitivity analysis

In this study, we use the one-factor-at-a-time (OFAT) method to explore the model's behavior and examine its sensitivity to changes in factors. We selected four factors for the analysis (Table 3). Given the stochastic nature of the model, it is necessary to conduct multiple runs to examine whether randomness (i.e., random seed) affects the model output. Therefore, each factor's change is analyzed from an average of 20 iterations with random seeds to reduce possible stochastic effects. We examine how changes in selected factors affect the number of adoptions across EER measures as well as the choice of the Consumat strategies in different districts. In this section, we demonstrate and discuss an example of Zuidoost and compare it with other districts different in their contexts. The results of the sensitivity analyses of the rest of the districts are offered in Appendix.

**Table 3:** Factors used for the OFAT sensitivity analysis.

Factors	Description	Scenarios
Electricity price	Change in electricity price (±15 cents per kWh)	0.082, 0.232 (base), 0.382
Gas price	Change in gas price (±40 cents per m^3^)	0.429, 0.829 (base), 1.229
Mean of energy prices’ uncertainty	Change in the mean of energy (electricity and gas) prices’ uncertainty	0.1, 0.2 (base), 0.3, 0.5, 0.7
List of similar neighbors	Change in the list of similar neighbors (original list based on location, age, and income; original list constrained additionally by education; removed age constraint from the original list; removed income constraint from the original list)	The original list (base), constraint_educ, remove_age, remove_income

### Electricity price

**Fig. 7:**
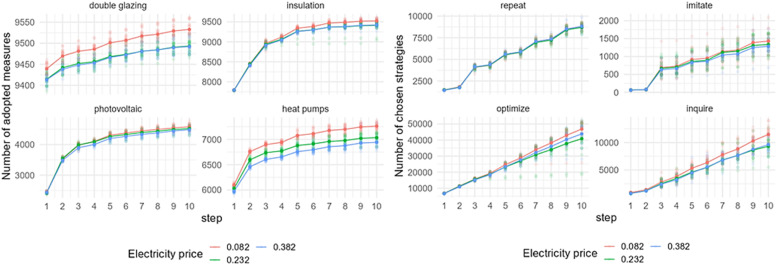
Sensitivity of model outputs (left: uptake of EER measures; right: behavioral strategies choice) based on the varied electricity prices in Zuidoost.

Fig. 7 shows the effect of an electricity price change on adopting EER measures and behavioral strategies choices in Zuidoost. The output shows the sensitivity of the model to electricity price changes.^6^ The adoption rate of EER measures varies depending on the electricity prices, with a higher number of measures being adopted when prices are reduced. The most significant differences in adoption rates are observed in the cases of double glazing and heat pumps. In terms of the chosen strategies by homeowners in Zuidoost, there is more variation with electricity price changes in the number of optimize and inquire strategies, with more of these strategies being chosen under the lower electricity price.

**Fig. 8:**
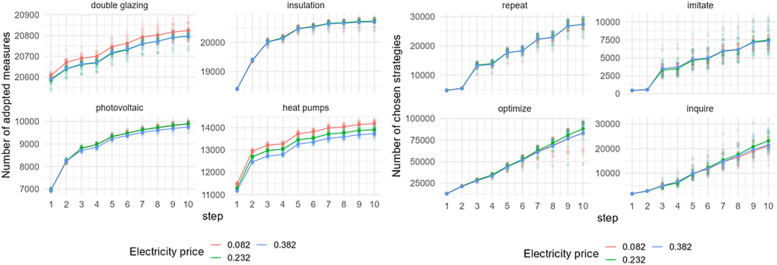
Sensitivity of model outputs (left: uptake of EER measures; right: behavioral strategies choice) based on the varied electricity prices in Oost.

The model sensitivity differs in the case of other Amsterdam's districts, even though the pattern of the EER uptake remains similar. In the case of Oost (Fig. 8), the adoption of double glazing and heat pumps is higher when electricity prices are lower, similar to Zuidoost. Although the difference appears more significant when comparing these two districts, it's essential to consider their varying scales when interpreting the results. However, regarding insulation and solar panels, the change in electricity prices has minimal impact on their uptake in Oost. The model output on chosen strategies in Oost is also sensitive to electricity price changes; however, it does not have a clear price scenario that would lead to a distinct output.

**Fig. 9:**
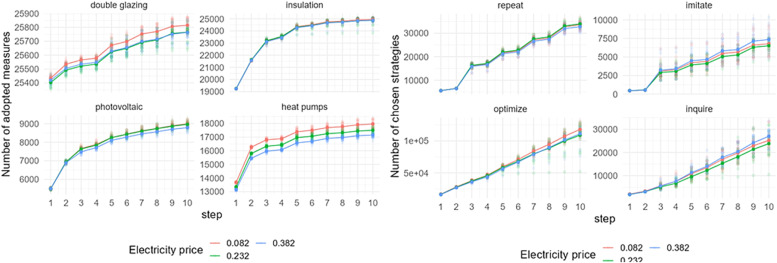
Sensitivity of model outputs (left: uptake of EER measures; right: behavioral strategies choice) based on the varied electricity prices in Zuid.

Another district that shows sensitivity to electricity price changes is Zuid (Fig. 9). Zuid is a larger district in terms of its population, and also in terms of the number of adopted measures. The pattern of the EER uptake in Zuid is similar to the patterns of the previous two districts. However, the number of chosen strategies slightly differs from the earlier examples. There are more homeowners that optimize under the lower electricity price, while fewer of them choose to inquire.

### Gas price

**Fig. 10:**
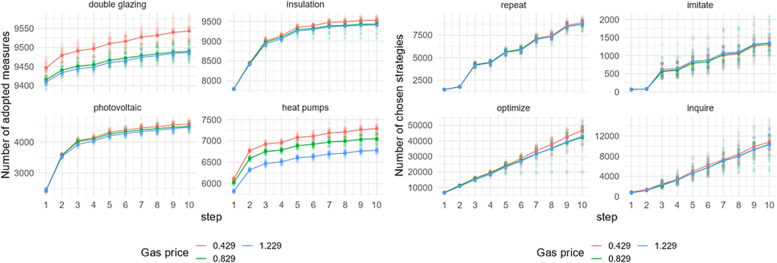
Sensitivity of model outputs (left: uptake of EER measures; right: behavioral strategies choice) based on the varied gas prices in Zuidoost.

Fig. 10 shows a gas price change's effect on adopting EER measures and behavioral strategies choices in Zuidoost. The adoption rate of the EER measures changes with the change in gas prices. As such, the model is the most sensitive to the changes in gas prices in terms of double glazing and heat pump uptake. The number of these two measures is increasing (to a different extent, though) with a lower gas price. In terms of behavioral strategy choices, there are some slight differences in the output. The differences concern the number of chosen optimize and inquire strategies, becoming more visible at the time step 6 (the year 2027) under the lower gas price.

**Fig. 11:**
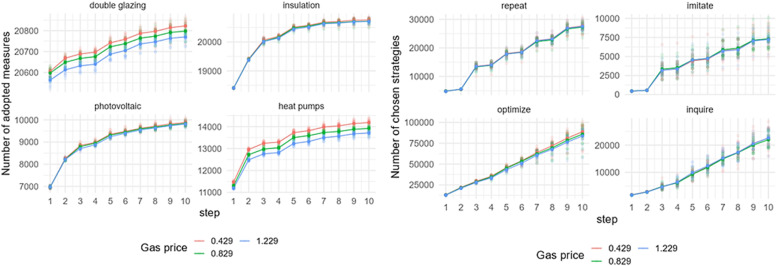
Sensitivity of model outputs (left: uptake of EER measures; right: behavioral strategies choice) based on the varied gas prices in Oost.

Similarly, in the case of Oost, when it comes to the adoption of double glazing and heat pumps, the model is particularly responsive to fluctuations in gas prices, showing high levels of sensitivity (Fig. 11). On the other hand, when the gas prices decrease, the difference in the adoption of double glazing in Oost is less pronounced. Regardless of how much gas prices change, the number of chosen strategies remains almost the same under all examined scenarios. Similar patterns of the model output have been observed in the case of Zuid (Fig. C.5).

### Mean of energy prices’ uncertainty

**Fig. 12:**
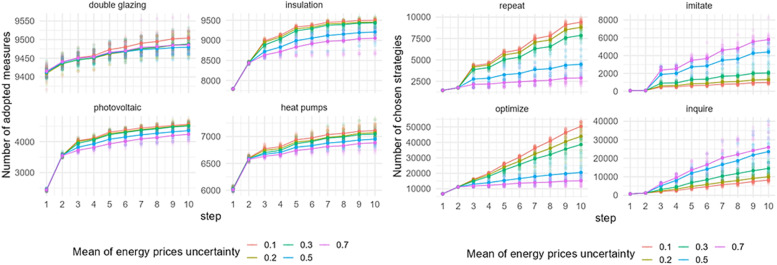
Sensitivity of model outputs (left: uptake of EER measures; right: behavioral strategies choice) based on the varied mean of energy prices’ uncertainty in Zuidoost.

Fig. 12 shows the effect of a change in the mean of energy prices’ uncertainty on the adoption of EER measures and behavioral strategies choice in Zuidoost. The model outputs are sensitive to a change in the mean of energy prices’ uncertainty. In general, the adoption of all EER measures is increasing with lower energy prices’ uncertainty, which means that the more certain the prices are, the more EER measures homeowners adopt. The change in the mean of energy prices’ uncertainty also affects the behavioral strategy choice. The number of repeat or optimize strategies chosen by homeowners increases with a lower mean of energy prices’ uncertainty, which aligns with the Consumat framework. With a much higher number of performed optimize strategy compared to the repeat strategy, the model output indicates a higher number of EER adoptions. In contrast, a higher mean of energy prices’ uncertainty implies more homeowners choosing to imitate or inquire. This means that with higher uncertainty, homeowners perform their strategy considering the behaviors of others. Similar patterns of the model sensitivity to the change in the mean of energy prices’ uncertainty are observed in Zuid (Fig. C.5).

**Fig. 13:**
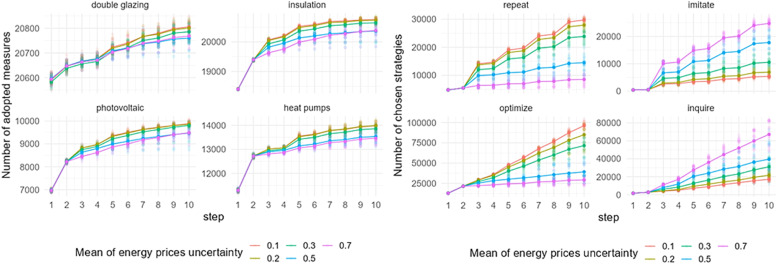
Sensitivity of model outputs (left: uptake of EER measures; right: behavioral strategies choice) based on the varied mean of energy prices’ uncertainty in Oost.

The model output based on the varied mean of energy prices’ uncertainty is slightly different for the case of Oost (Fig. 13), though the pattern of the uptake is similar to the pattern that observed in other districts when varying this factor. In Oost, there is no observable difference in the uptake of all EER measures under two uncertainty levels – 0.1 and 0.2. Also, homeowners choose to inquire more under the highest level of uncertainty examined, which is different for other districts.

### List of similar neighbors

**Fig. 14:**
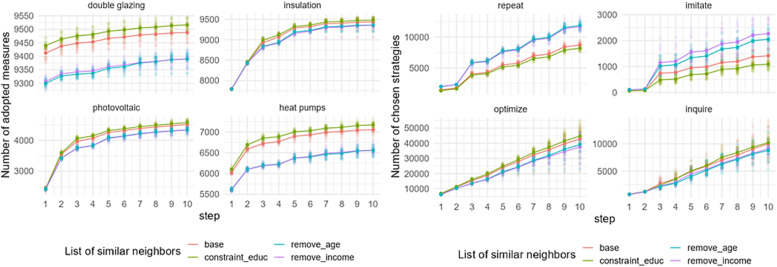
Sensitivity of model outputs (left: uptake of EER measures; right: behavioral strategies choice) based on the varied list of similar neighbors in Zuidoost.

Fig. 14 shows the effect of a change in the list of similar neighbors on the uptake of EER measures in Zuidoost. We examined two scenarios removing one constraint at a time (age – “remove_age” and income – “remove_income”) and adding one constraint (education – “constraint_educ”). Removing constraints expands the list of similar neighbors with whom homeowners interact while adding a constraint shortens this contact list. The model output is sensitive to a change in the list of similar neighbors. The output shows that interacting with more similar neighbors (based on location, age, income, and education level) increases the adoption of all EER measures. In contrast, expanding the similar neighbors’ list decreases the EER adoption rate. Under both scenarios, the difference is larger for double glazing and heat pump adoption.

The change in the list of similar neighbors also affects the behavioral strategy choice. The output shows that homeowners choose one of the reasoned strategies under the scenarios when homeowners interact with more similar neighbors (base and constraint_educ). As the reasoned strategies imply adopting the measures, the list of more similar neighbors is the best option for the EER uptake. In contrast, with the expanded similar neighbors’ list, homeowners choose one of the automated strategies, such as repeat and imitate. Similar results of the EER uptake and chosen strategies based on the varied list of similar neighbors are observed in Oost (Fig. C.6).

**Fig. 15:**
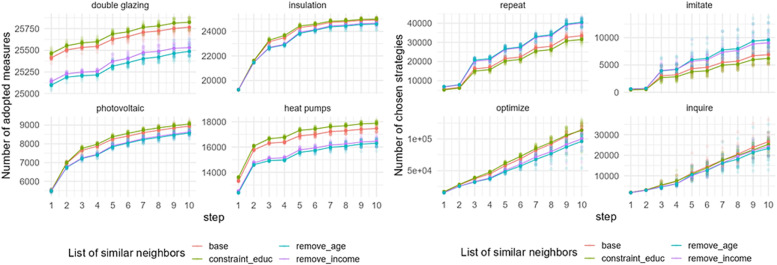
Sensitivity of model outputs (left: uptake of EER measures; right: behavioral strategies choice) based on the varied list of similar neighbors in Zuid.

Sensitivity of the model outputs in Zuid is marginally different compared to other districts (Fig. 15). This difference is caused by the difference in the population size of the districts, in general. That is, the patterns observed in Fig. 14, Fig. 15 look similar, but should be interpreted differently as their scales differ. Additionally, it is interesting to notice that when homeowners interact with similar neighbors based on location and age, the uptake of double glazing and heat pump is slightly higher than when they interact with similar neighbors based on location and income.

In summary, based on the sensitivity analysis, it is evident that changes in factors’ values affect the model output. The results of the sensitivity analyses across the rest of the districts show various model patterns in terms of the EER uptake and behavioral strategy choice that can be found in the Appendix. Overall, the obtained results from the sensitivity analysis allowed us to explore the model's behavior and potential outputs under the scenarios varying one factor at a time.

#### Interaction experiment

In addition to the OFAT method that enables exploring and understanding the model's behavior, examining possible interaction effects is useful [43]. One of the commonly used methods for testing the interaction is a standardized linear regression [44]. However, the Energy Pro-model is limited to 10 time steps, which does not allow for producing enough output to examine it in the regression. Therefore, in this study, to examine the existence of interactions in the model, we simply vary two factors at a time. It is a quick-and-dirty way of studying the output and gaining more insights into the model's behavior.

**Fig. 16:**
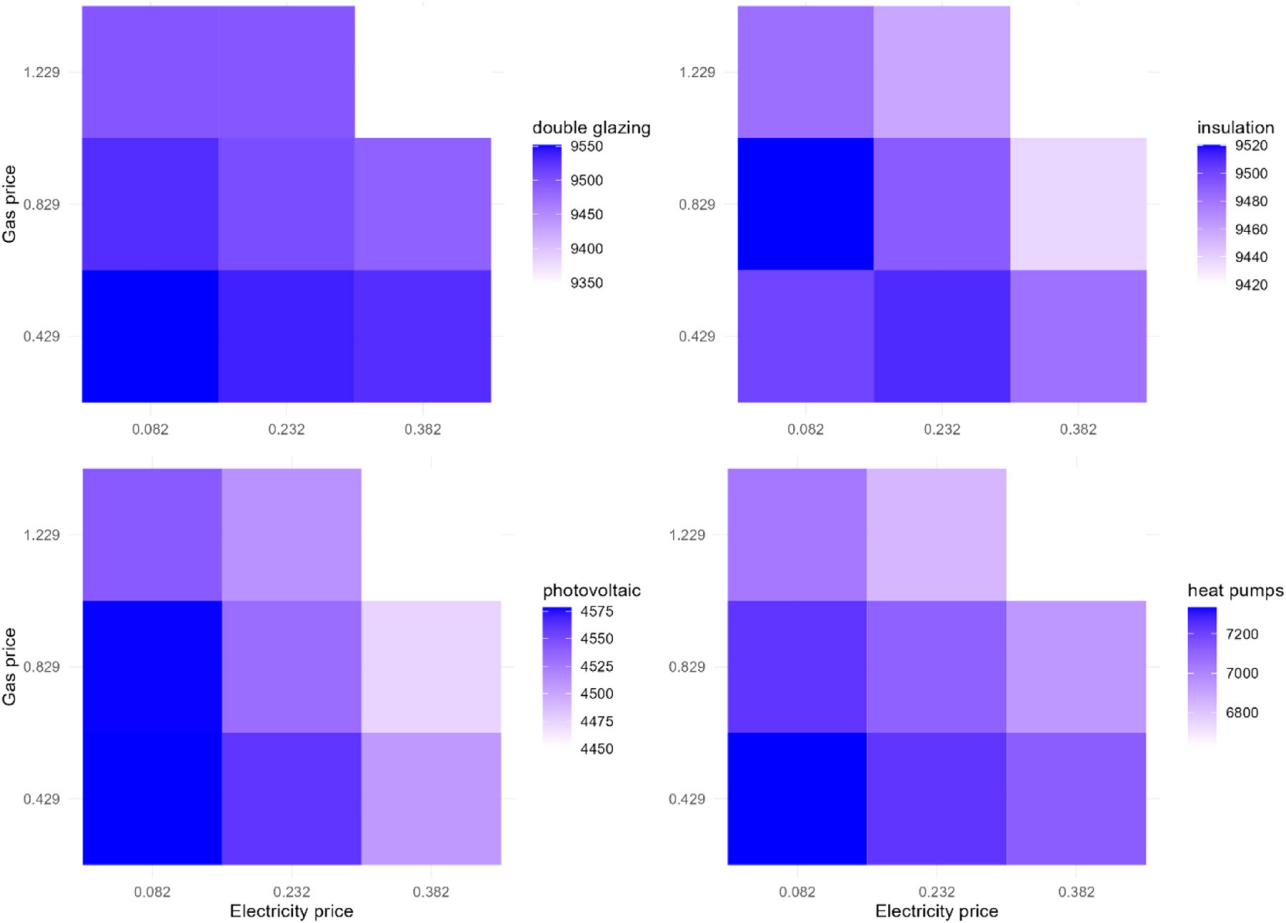
The effect of the interaction of gas and electricity price changes on the EER uptake in Zuidoost.

To explore the interaction, we vary electricity and gas prices and examine their interaction effect on the EER uptake. Fig. 16 demonstrates the effect of the interaction of gas and electricity price changes on the EER uptake in Zuidoost. This experiment shows that the highest uptake of double glazing, heat pumps, and solar panels occurs under the scenario when the electricity price is reduced by 15 euro cents per kWh and the gas price is reduced by 40 euro cents per m^3^. Additionally, there might be high uptake of solar panels under the base gas price and lower electricity price (reduced by 15 euro cents). This also holds for the case of insulation adoption rate. It can also be noticed that there are less favorable uptake scenarios under the electricity price increase by 15 euro cents per kWh. Overall, there are multiple different energy price change scenarios under which the EER uptake can potentially be high in Zuidoost.

**Fig. 17:**
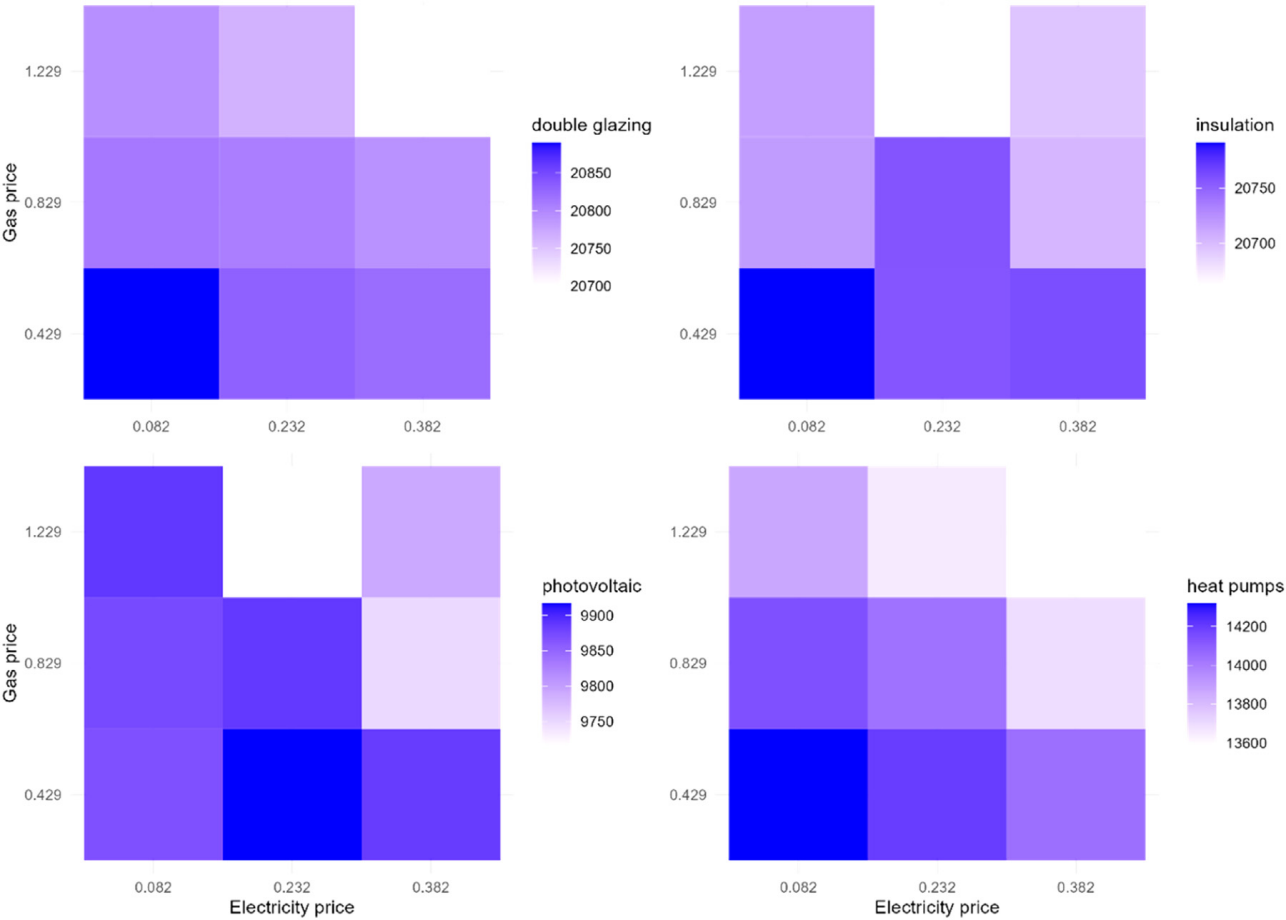
The effect of the interaction of gas and electricity price changes on the EER uptake in Oost.

In contrast, there are less scenarios that are favorable for the EER uptake in Oost in general (Fig. 17). In the case of this district, there are more scenarios of lower EER uptake under the higher gas price that increases by 40 euro cents per m^3^, whereas the Zuidoost example shows that this occurs under the highest electricity price with an increase by 15 euro cents per kWh. The highest adoption rate of double glazing, insulation, and heat pumps might be under the lowest tested electricity and gas prices that are reduced by 15 euro cents per kWh and 40 euro cents per m^3^, respectively. However, in terms of solar panels, the highest adoption rate is feasible with the base electricity price and lower gas price (reduced by 40 euro cents).

**Fig. 18:**
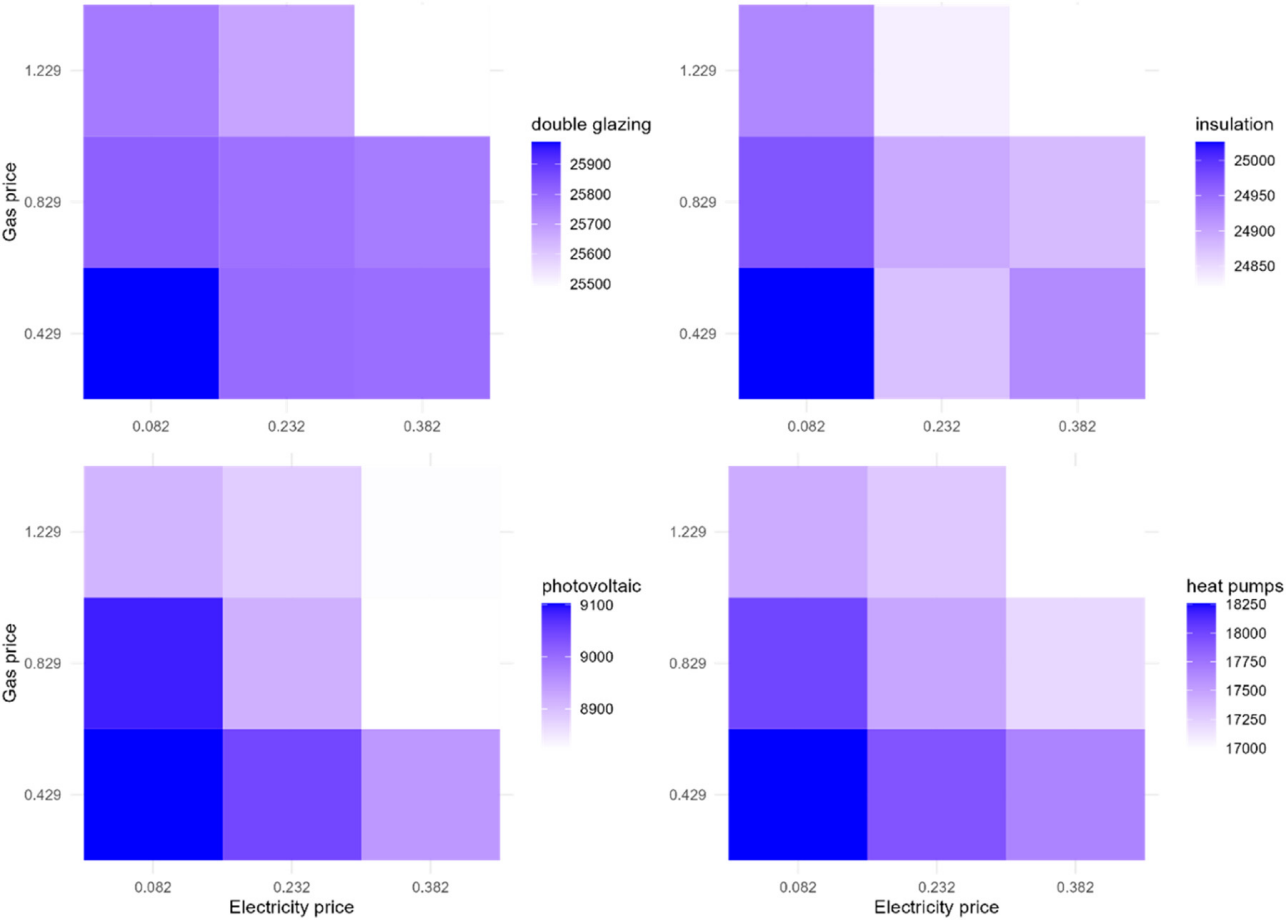
The effect of the interaction of gas and electricity price changes on the EER uptake in Zuid.

In Zuid, there are even less scenarios that are favorable for the EER uptake (Fig. 18). The highest rate of the EER uptake occurs under the lowest electricity and gas prices in this district (reduced by 15 euro cents per kWh and 40 euro cents per m^3^, respectively).

This interaction experiment provides additional insights on the model behavior and can also be useful for designing potential policy interventions taking into account the contextual differences of the districts. The interaction experiments results of the rest of the districts can be found in Appendix. We did not conduct other interaction experiments varying two factors as this is not as accurate to examine the existence of interactions as it could be when using the regression analysis.

### Model calibration and validation

#### Model calibration

Calibrating the model for the simulation period of 2021–2030 is a challenging task since it involves projecting into the future. As such, it is difficult to obtain calibration data that reflects the actual conditions of the simulation period. Despite this challenge, having access to relevant data can help partially calibrate the model. Satellite images for PVs in Amsterdam can be one of the important sources for calibrating this model. However, the difficulty arises from the absence of satellite images beyond 2021. This issue underscores the importance of the government's attention to the matter. Access to data would facilitate research and enhance the accuracy of policymaking. A possible solution is to mandate annual reporting from homeowners-adopters on the measures taken, if any. Nonetheless, it is essential to recognize that the model's projections are subject to uncertainties and should be interpreted with caution.

#### Model validation

This study heavily relies on expert validation, given the specificity of the model's simulation period discussed above. The process of expert validation involved interviewing energy experts.^7^ The experts participated in a structured interview process that involved several steps. Firstly, they were presented with a general question regarding their expectations for the uptake of the four EER measures. Secondly, they were asked to reflect on the factors they believed were the most important for driving EER uptake. Thirdly, they were shown a model simulation and asked to evaluate whether the outputs were consistent with their expectations. Fourthly, they were asked to consider the results of sensitivity experiments. Fifthly, they were asked to reflect on potential differences in EER uptake across different districts. Finally, each expert was asked to propose policy interventions that could be instrumental in accelerating the energy transition in Amsterdam by 2030. Each expert focused on a specific district in their analysis.

#### Expectations about the EER uptake

The experts encountered challenges when trying to reflect on their expectations for EER uptake, with only one common expectation emerging among them. Specifically, all three experts agreed that double glazing and insulation were likely to see higher uptake due to their relative ease of adoption in the city. To validate the overall expectations for EER uptake, one of the experts suggested referring to official documents outlining Amsterdam's future vision and expectations for energy transition, such as the Roadmap of Amsterdam [20] and the Heat Transition Vision of Amsterdam [45]. However, these documents were found to lack specific targets for residential EER uptake in the city, focusing instead on broader goals for the energy transition. As a result, it is challenging to form a clear picture of expected EER uptake in Amsterdam.

### The most important factors affecting the EER uptake

According to the opinions of the three experts, gas and electricity prices are the most significant factors that influence the uptake of energy efficiency measures. They also point out that energy price uncertainty is closely related to changes in energy prices and can significantly impact households' decision-making. Furthermore, two energy experts stress the significance of social influence, especially that of neighbors, in promoting the adoption of visible measures such as PV. They note that in smaller neighborhoods, people tend to be more closely acquainted, and information can spread quickly through word of mouth. The experts' views support the meaningfulness of the factors chosen for the OFAT sensitivity analyses. Additionally, the experts emphasize that the combination of gas and electricity prices is expected to have a more substantial effect on homeowners' adoption decisions.

### The model outputs

The experts found the model outputs to be both meaningful and insightful. During their feedback sessions, each expert concentrated on a specific district, acknowledging the variations in uptake within the areas they examined. They attributed most of these differences to differences in households and dwelling characteristics. The experts further observed that these variations occurred at the neighborhood level, emphasizing the importance of narrowing the research focus to gain a more comprehensive understanding of household decision-making.

During the discussion of the model outputs, the experts raised two points of concern. The first was a higher uptake of heat pumps compared to solar panels in the Centrum district. Two experts noted that the adoption of heat pumps was expected to be the slowest among the four EER measures in this area due to space constraints and the complexity of heat pump installations in the majority of monumental buildings found in Centrum.

The experts also noted a significant uptake of the EER measures in the first time step (the year 2022), which is due to the low level of need satisfaction among homeowners caused by the high LNS_min_ threshold. The model includes a slider that allows for the exploration of different scenarios by adjusting this variable. When the LNS_min_ is lower, the overall uptake of all measures is also lower, particularly the adoption of heat pumps. However, one expert highlighted that the drastic uptake observed in the model may not always be due to the level of need satisfaction among households, but rather to various macro-level factors. Therefore, the model's outputs should be considered alongside other contextual factors to gain a better understanding of the dynamics driving the uptake of EER measures.

On the other hand, two expert who focused on the Centrum district emphasized that the level of need satisfaction is a crucial factor in the adoption of EER measures in this particular area. They noted that the majority of homeowners in Centrum are high-income residents who prioritize their comfort and are willing to invest significant amounts of money to enhance it. Therefore, in the Centrum model simulation, adjusting the LNS_min_ using the slider and observing how it affects the output is meaningful. During the interview, we ran an experiment to determine whether the model output would change and whether it was significant. As anticipated by the two experts, the model output in Centrum was sensitive to a lower LNS_min_, resulting in a lower uptake of heat pumps and a higher uptake of solar panels, which has reflected reality better.

### Sensitivity experiments outputs

The experts have emphasized that sensitivity experiments provide valuable insights into the model's behavior. During the OFAT sensitivity analysis, it was observed that the model is sensitive to changes in all selected variables. However, in the case of energy price changes, the model output was found to be more sensitive to the gas price change, which all experts agree upon. The experts have also unanimously agreed that the gas price is and will remain the most critical factor affecting the adoption rate of EER measures in the Netherlands. Moreover, the experts have evaluated the effect of mean energy price uncertainty on the EER adoption rate, and they find it reasonable. It has been observed that homeowners with less uncertainty tend to adopt EER measures more actively, while they also choose to repeat or optimize more often. Additionally, experts have noticed an interesting pattern in the adoption rate concerning varying lists of similar neighbors. For reasoned behavior, it matters that the list of similar neighbors is more constrained. This means that the more similar the neighbors are, the more likely homeowners are to adopt the measures. However, for automated behaviors, homeowners tend to consider overall neighbors in their surroundings more than just similar ones.

Additionally, we asked the experts to identify the key combination of factors that would influence EER uptake before presenting the results of the interaction experiment. One of the experts pointed out that the combination of electricity and gas prices was critical in this regard. Subsequently, we demonstrated the output of the interaction experiment, and the experts acknowledged that the findings were significant and reflective of reality. However, the experts cautioned that the interpretation should be made carefully, as the interaction experiment only shows the overall EER adoption value at the end of the simulation period, which is subject to uncertainty.

### Differences across the districts

According to all three experts, there are significant differences in EER uptake across the districts in Amsterdam. The experts caution that the results should be interpreted in the context of households' and districts' characteristics. One expert emphasizes that the most critical factors contributing to differences across districts are the type of dwelling, location (part of the city where households live), the number of people with house ownership status in the district, households' income, and available subsidies for EER adoption. The experts also agree that differences exist within districts, and a more detailed investigation at the neighborhood level is necessary for further studies, which would also help to validate the model more comprehensively.

### Policy intervention scenarios

We consulted with experts to identify potential policy interventions to explore in the next phase of this research. The experts agreed that some policies should be implemented city-wide, while others should be tailored to the unique context of each district. The experts identified controlling residential gas consumption through a gas tax as the most critical policy intervention for Amsterdam's energy transition. Additionally, they recommended exploring policy scenarios such as providing more subsidies for adopting EER measures and increasing public awareness of their importance. However, the experts cautioned that EER measures are complex and can demotivate households from adopting them. Thus, easing EER adoption regulations and related policies will also be crucial. The experts also emphasized the importance of ensuring that all population groups can participate in Amsterdam's residential energy transition without exclusion.

### Using and adapting the model

The first step when running the model is to select a district we are interested in, using the chooser (1). Second, we adjust the model parameters for the desired analysis (2). The parameters that can be adjusted include the mean of the energy price uncertainty and the standard deviation of the uncertainty of individual owner-occupied households by dragging the sliders. The two monitors show the electricity and gas price changes over the simulation time. It is also possible to switch the collective decision-making of homeowners on PV adoption, the new random seed, the LNS_min_ adjustment, whether only owners make EER decisions or both owners and tenants, and whether multi-apartment buildings consist of only owner-occupied apartments. Some of these parameters are used for sensitivity analysis. After the parameters are set, we load the GIS data with the default settings, including households’ socio-demographic and dwelling characteristics of the chosen district (3).

Depending on the districts' population size, the model takes about 7–8 s to load the settings. Finally, the model can be run either for the whole simulation time or for one time step using the buttons (4). All the settings can also be removed, and the electricity and gas prices can be set to default values, clearing the previously loaded values based on parameters’ adjustments (5). Using the buttons underneath the worldview, one can load distributions of different EER measures’ adoption or their average adoption rates. A color palette ranging from red to green is chosen to represent the adoption level, where red means no adoptions are made and green means all measures are adopted. On the right side of the worldview, several monitors demonstrate some of the set characteristics’ of the chosen district's population and calculated values (e.g., average electricity consumption per household).

The ENERGY Pro-model can be adapted for other places and other time periods, as well as can be expanded by adding other energy system elements such as electric mobility. Users should take care to verify and validate the model after making changes. We recommend using R programming language to analyze such a large model as it speeds up and automatizes the process.

## Limitations

This study has the following limitations. First, the model lacks empirical validation due to limited data availability. This can be improved by collecting real-time data, e.g., mandating that households report to the municipality about adopted measures and using satellite images (though applicable only to solar panels). This way, the local authorities would be aware of up-to-date information on residential EER adoptions. Second, a lack of socio-psychological and cultural data hindered our understanding of “why” households make certain decisions. Incorporating these important factors would enable future research to gain more in-depth knowledge about households’ behavior. Third, the coarse time granularity of the simulation might have hindered capturing the emergent behavior of the modeled system. Increasing the number of time steps in future simulations could enable researchers to collect more data to improve the sensitivity analysis and better understand the emergent phenomenon.

In addition, future research can benefit from a more granular analysis (i.e., focusing on one neighborhood/district), a reduced number of investigated measures, and incorporating other actors (e.g., landlords, local authorities, energy companies). Although this suggested change in the model's construct will serve other research purposes, it can offer a deeper understanding of the local energy transition.

## Ethics statements

Not applicable.

## Software and data availability

This study uses NetLogo 6.3.0 (available from: https://ccl.northwestern.edu/netlogo/) and R “ipfp” and “mice” packages (available from: https://www.r-project.org).

The model code, its associated files, and the R script for creating the synthetic population are available from https://github.com/erkinaiderkenbaeva/Energy-Pro-model.

The synthetic population dataset created is available from DANS Data Station Social Sciences and Humanities at https://doi.org/10.17026/SS/LUV9KW [46].

## CRediT authorship contribution statement

**Erkinai Derkenbaeva:** Conceptualization, Methodology, Data curation, Software, Investigation, Formal analysis, Writing – original draft, Writing – review & editing, Validation, Visualization. **Gert Jan Hofstede:** Conceptualization, Methodology, Writing – review & editing, Supervision, Formal analysis, Visualization. **Eveline van Leeuwen:** Conceptualization, Methodology, Data curation, Supervision, Funding acquisition. **Solmaria Halleck Vega:** Conceptualization, Methodology, Writing – review & editing, Supervision. **Juriaan Wolfers:** Investigation, Software.

## Declaration of competing interest

The authors declare that they have no known competing financial interests or personal relationships that could have appeared to influence the work reported in this paper.

## Data Availability

The authors have shared the links to data and code

## Appendix Supplementary materials

Image, application 1

## Footnotes

1 Dutch automated system of Basic Registration Addresses and Buildings

2 In this model, there are 10 000 cells (100*100) in total, 6 379 of which are within the borders of the Zuidoost district and 1 846 cells occupied by households. The total population of Zuidoost is 41 652 households (as such, 41 652 / 1 846 cells = 23 households per cell). Considering this district's surface area is about 22 km^2^, each cell will represent 0.003 km^2^ or 3000 m^2^ (i.e., 22 km^2^ / 6 379 m^2^).

3 There were 476 008 households in Amsterdam based on Census data in 2021.

4 In this study, neighbors are chosen as a reference group based on previous studies’ findings that proved the importance of the neighborhood and neighbors’ impact on EER adoption [47].

5 This means a household is unable to afford adequate access to energy services necessary for basic energy needs.

6 It should be noted that the scale of the number of adoptions differs across the four EER measures; therefore, the adoption output of each measure should be carefully interpreted.

7 Energy experts included a strategic advisor at the Sustainability department of the Municipality of Amsterdam, a senior assistant professor at Wageningen University who previously worked as a researcher at the energy project in Zuidoost district, and a research fellow at Amsterdam Institute for Advanced Metropolitan Solutions.
