# Supplementary material for: ENERGY Pro: Spatially explicit agent-based model on achieving positive energy districts
Source: MethodsX. 2024 May 28;12:102779. doi: 10.1016/j.mex.2024.102779 (PMC11222806; doi:10.1016/j.mex.2024.102779)
Supplement: Supplementary file 1 [file mmc1.docx]

**Supplementary material *and/or* additional information: Appendix**

Table A. 1. The main global variables

| Global variables | Description |
| --- | --- |
| Electricity price | Electricity price (2021 and 2022, respectively) = 0.23 and 0.35 euros per kWh. For the rest of the simulation period, the electricity price fluctuates based on price uncertainty |
| Gas price | Gas price (2021 and 2022, respectively) = 0.83 and 1.63 euros per m^3^. For the rest of the simulation period, the gas price fluctuates based on price uncertainty |
| Uncertainty tolerance | Uncertainty tolerance is normally distributed with a mean of 0.5 with a standard deviation of 0.1 |
| Energy price uncertainty | Starting from 2023, energy prices fluctuate based on uncertainty (between 0 and 1) |
| Overview strategies | Global lists of the total number of Consumat strategies implemented by: (1) all households, (2) owners, (3) tenants, (4) per time step |
| Overview investments | Global lists of the total number of four EER measures adopted by: (1) all households, (2) owners, (3) tenants, (4) per time step |
| Residential carbon emissions (Amsterdam) | Residential carbon emissions in Amsterdam in 1990 and 2021 (tons), according to the consumption approach (sources: RVO, Regional Climate Monitor).  Gas carbon emissions (1990): 891 928  Gas carbon emissions (2021): 666 932  Electricity carbon emissions (1990): 407 000  Electricity carbon emissions (2021): 288 748 |
| Residential carbon emissions-saved | The difference in electricity and gas carbon emissions calculated between 2021-2030 with and without EER adoption |
| Energy emission factor in 2021 (Amsterdam) | Electricity emission factor (ton/kWh) = 0.000315, gas emission factor (ton/m^3^) = 0.001785 |

Table B. 1. Synthetic population validation: goodness-of-fit measures output

| Goodness-of-fit measures | Description | Output |
| --- | --- | --- |
| 1. Fit between constraints and estimates | Correlation between values of constraints and corresponding simulated values | Correlation = 0.9962695 |
| 1. Correlation for each neighborhood | Correlation between values of constraints and corresponding simulated values per neighborhood identifies the representativeness of neighborhoods | Acceptable min. correlation = 0.9.  Wijk WK036392 has the worst correlation (cor = 0.8) |
| 1. Number of districts and neighborhoods created | In 2021, there were 8 districts and 99 neighborhoods in Amsterdam | 1 district and 5 neighborhoods are omitted:  District: Westpoort  Neighborhoods:  NA’s – WK036311 (Nieuw-West), WK036350 (Oost), WK036372 (Noord); worst correlation – WK036392 (Zuidoost), worst fit – WK036310 (Westpoort) |
| 1. Standardized absolute error (relative error) | Total absolute error is a difference between the observed and simulated population divided by the total population multiplied by the number of constraints | RE = 3.6% |
| 1. Distribution of household size categories per district | Share of different types of households in terms of their size across the districts | The distribution output is offered in the Table B. 2 |

Table B. 2. Distribution of household size categories per district

| District | Share of 1-person households | | Share of 2-people households | | Share of 3(or more)-people households | |
| --- | --- | --- | --- | --- | --- | --- |
|  | Census | estimated | Census | estimated | Census | estimated |
| Centrum | 62% | 62% | 23% | 26% | 15% | 12% |
| Nieuw-West | 48% | 47% | 20% | 24% | 32% | 28% |
| Noord | 47% | 47% | 20% | 25% | 33% | 28% |
| Oost | 52% | 52% | 22% | 26% | 27% | 22% |
| West | 56% | 56% | 23% | 27% | 21% | 17% |
| Westpoort | 84% | 17% | 7% | 83% | 9% | 0% |
| Zuid | 55% | 54% | 24% | 27% | 21% | 18% |
| Zuidoost | 54% | 53% | 15% | 23% | 31% | 24% |


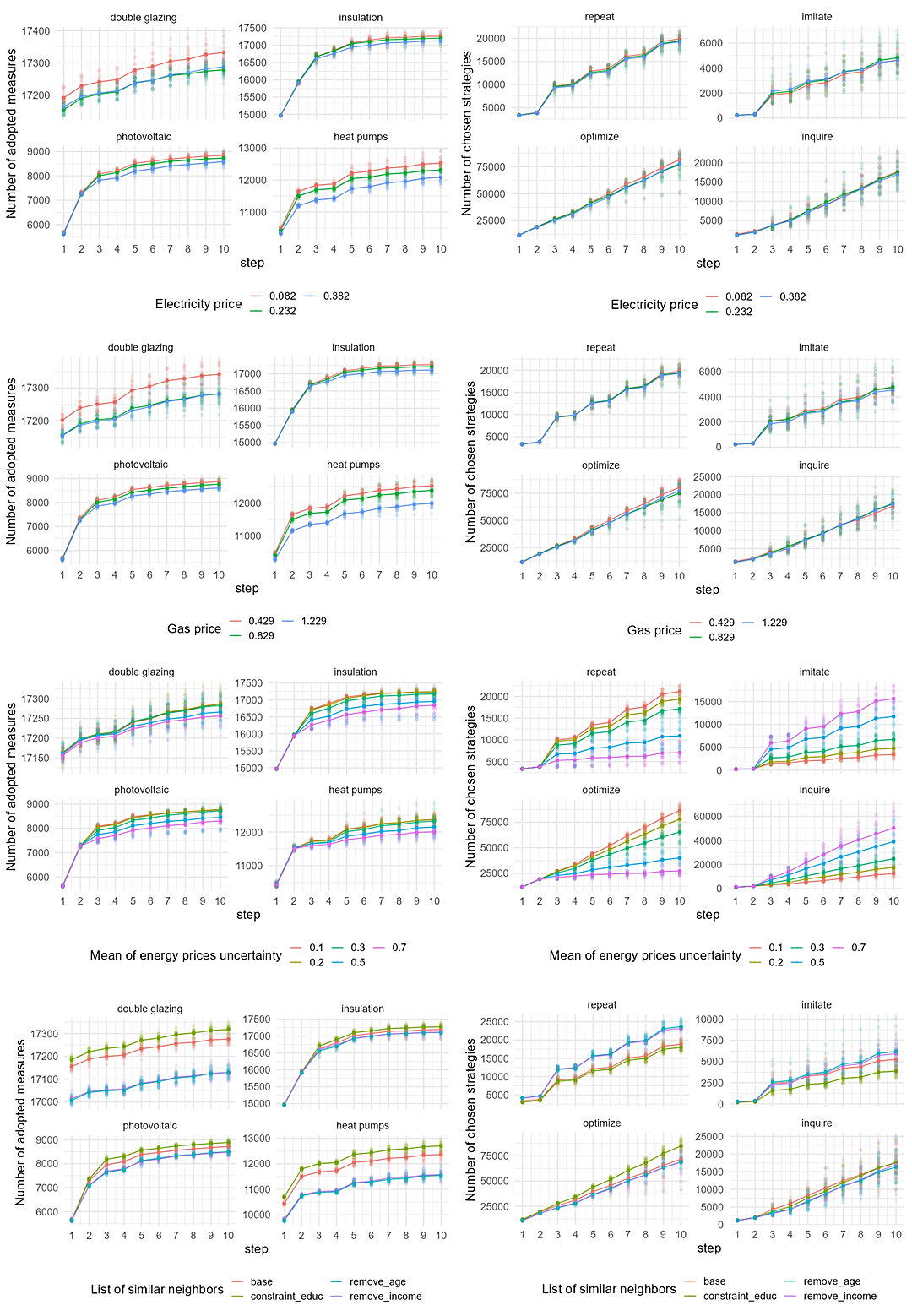


Figure C. 1. Sensitivity of model outputs based on the varied factors’ scenarios in Nieuw-West


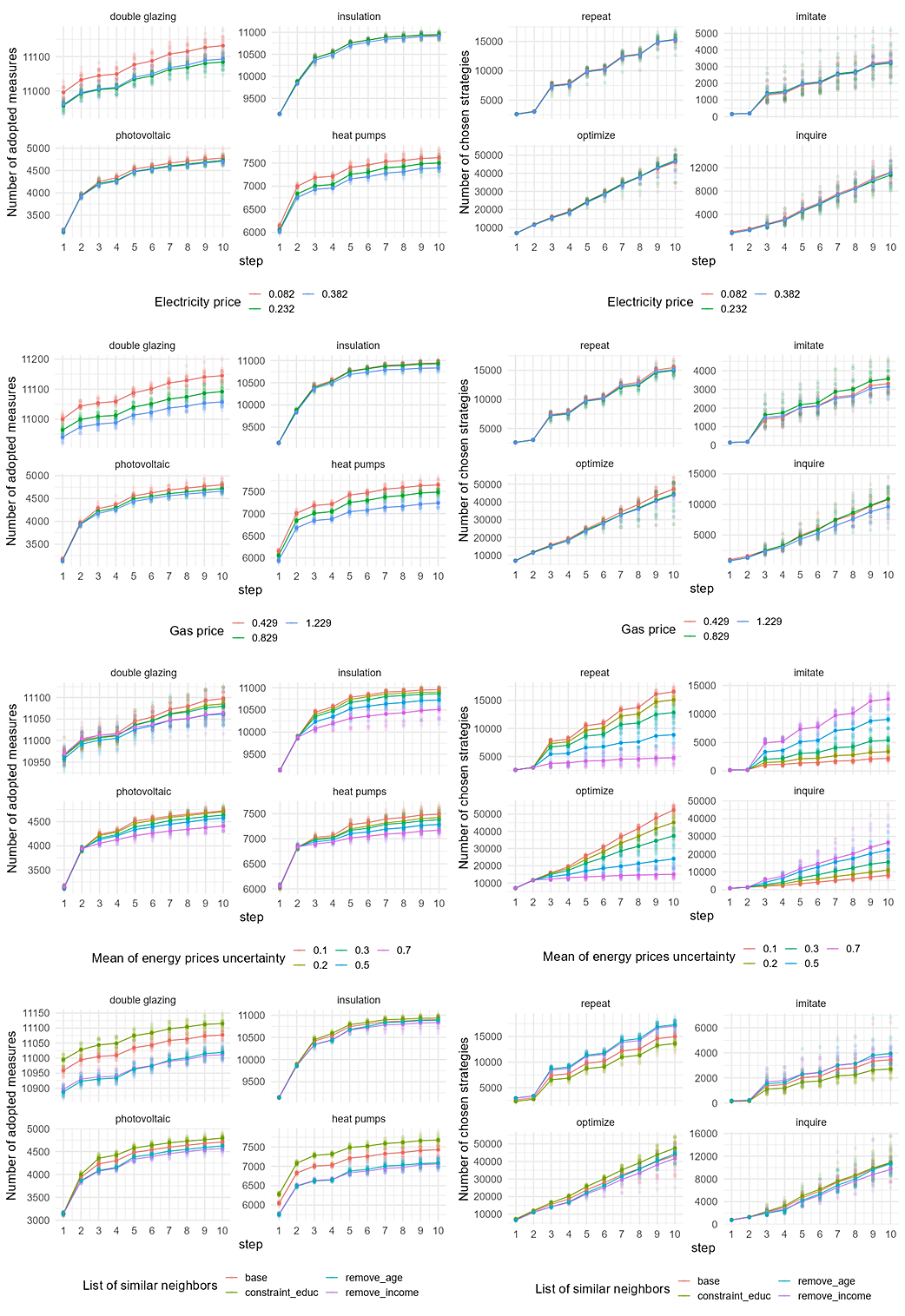


*Figure C. 2. Sensitivity of model outputs based on the varied factors’ scenarios in Noord*


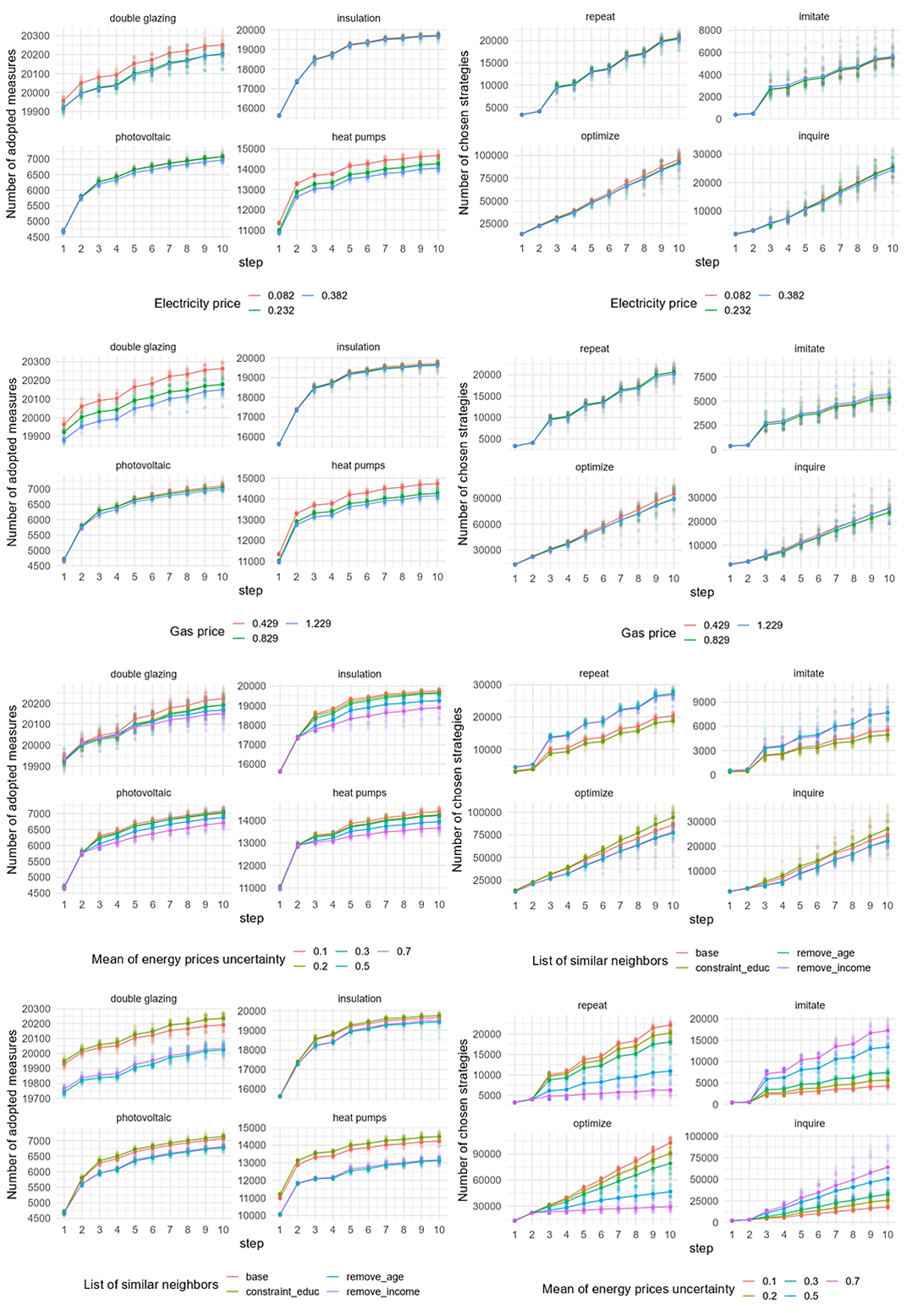


*Figure C. 3. Sensitivity of model outputs based on the varied factors’ scenarios in West*


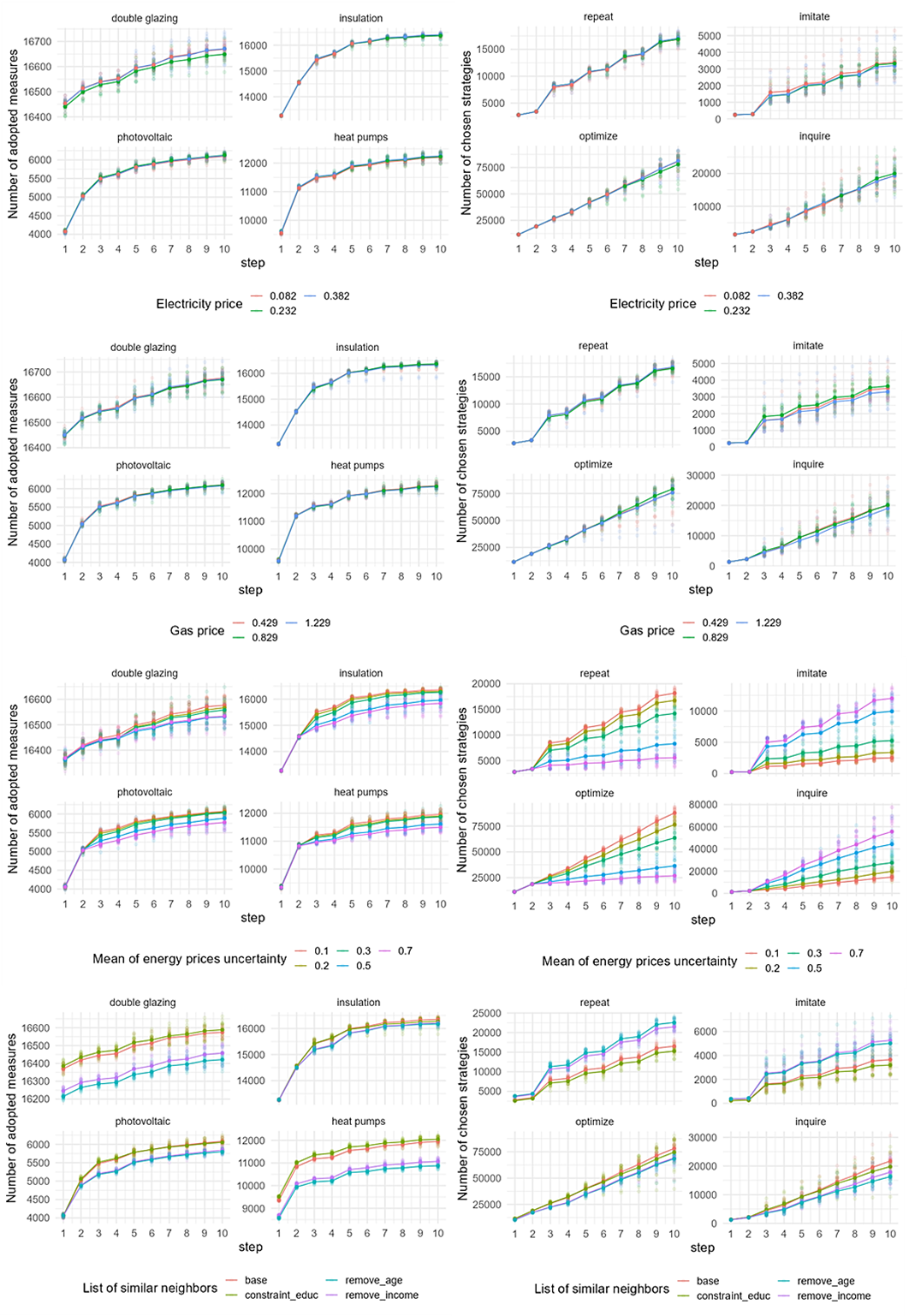


Figure C. 4. Sensitivity of model outputs based on the varied factors’ scenarios in Centrum


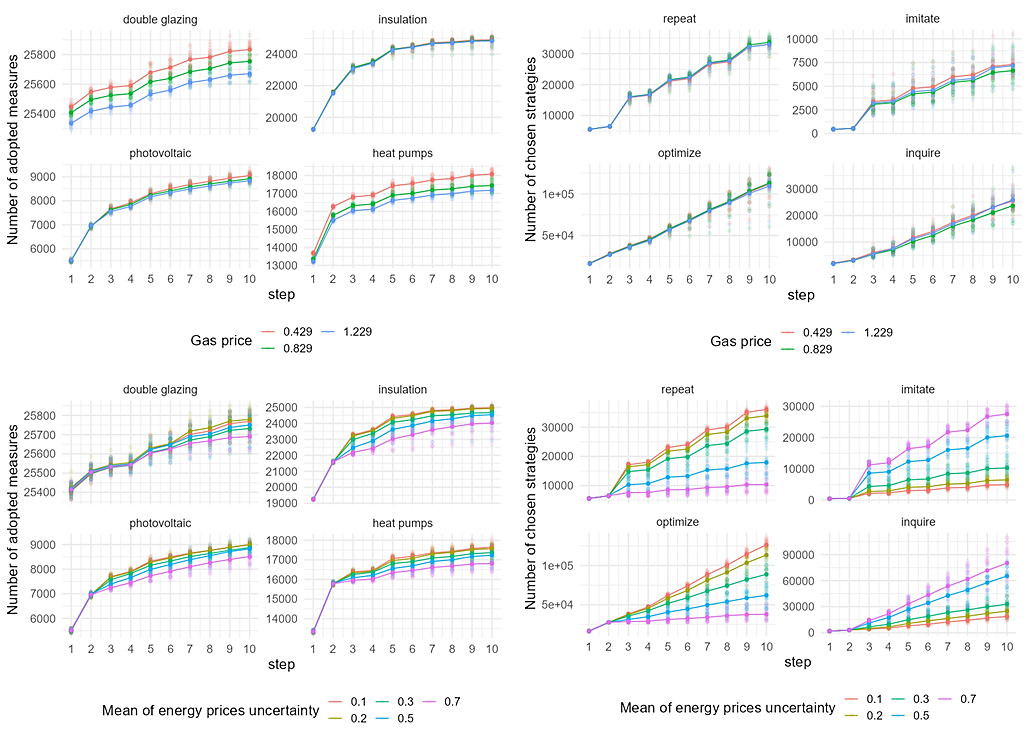


Figure C. 5. Sensitivity of model outputs based on the varied factors’ scenarios in Zuid


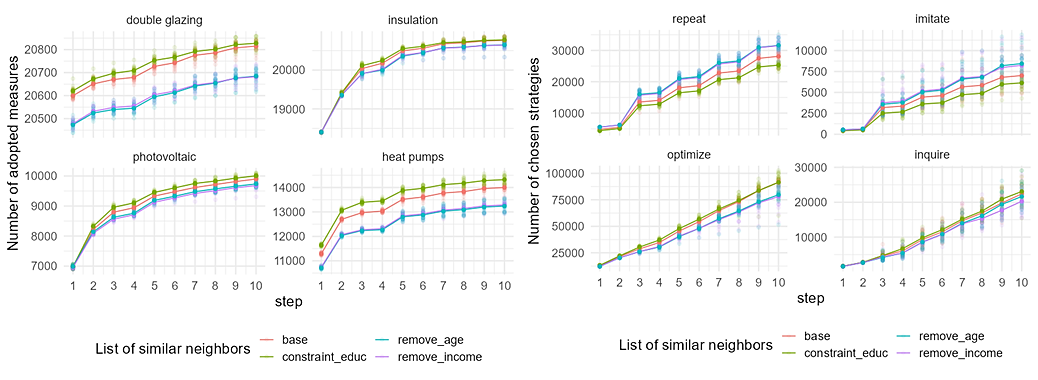


Figure C. 6. Sensitivity of model outputs based on the varied factors’ scenarios in Oost


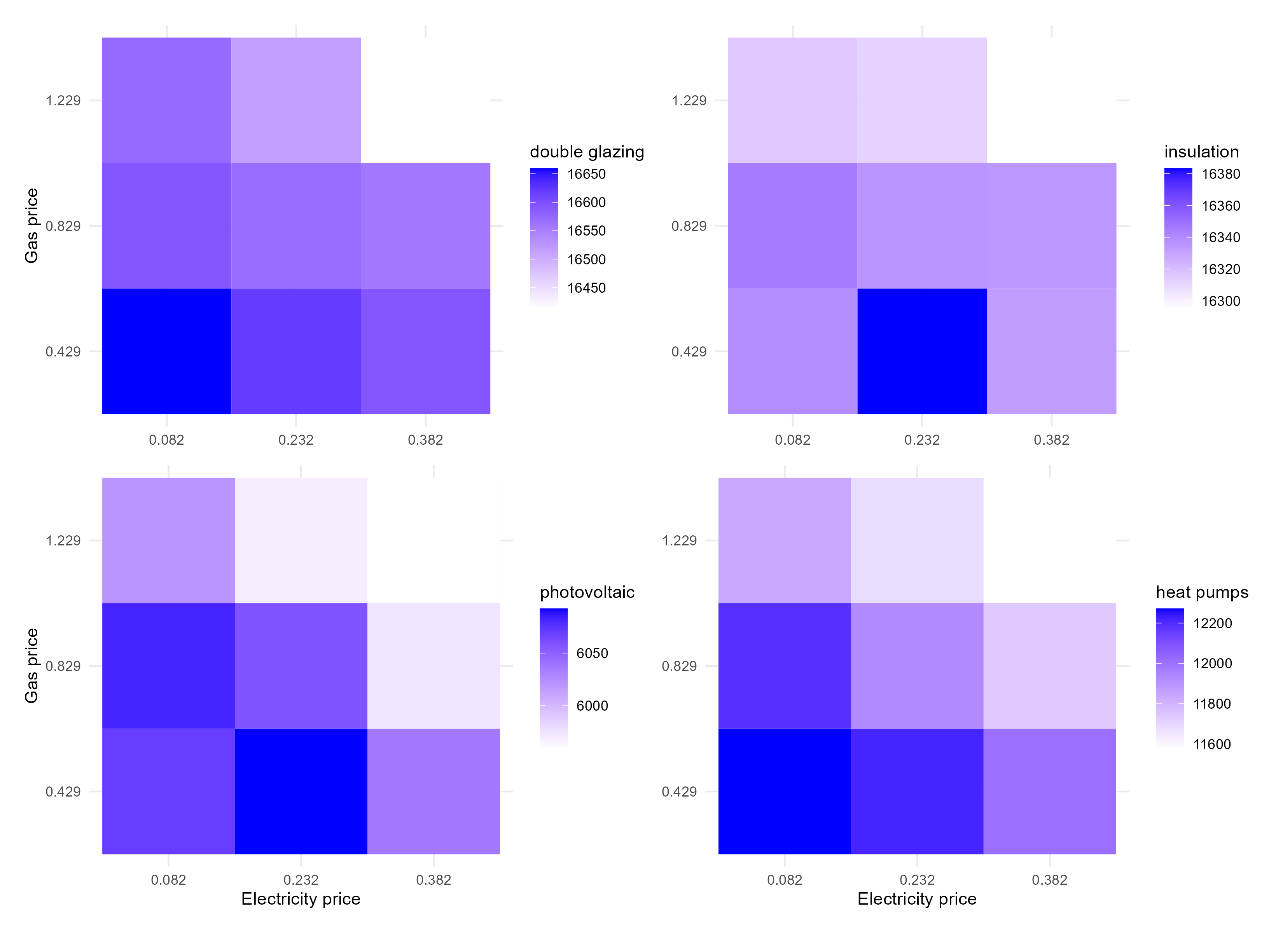


Figure C. 7. The effect of the interaction of gas and electricity price changes on the EER uptake in Centrum


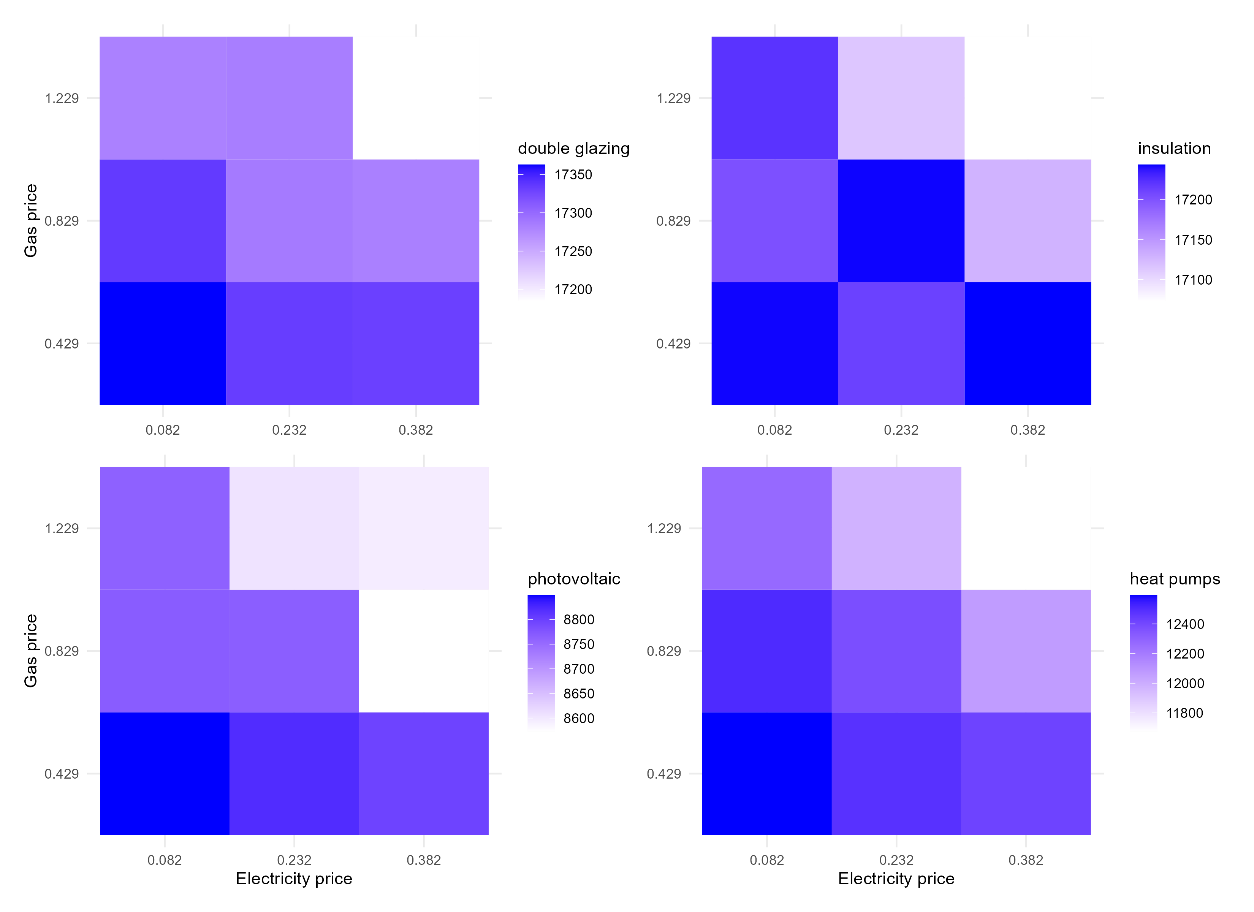


Figure C. 8. The effect of the interaction of gas and electricity price changes on the EER uptake in Nieuw-West


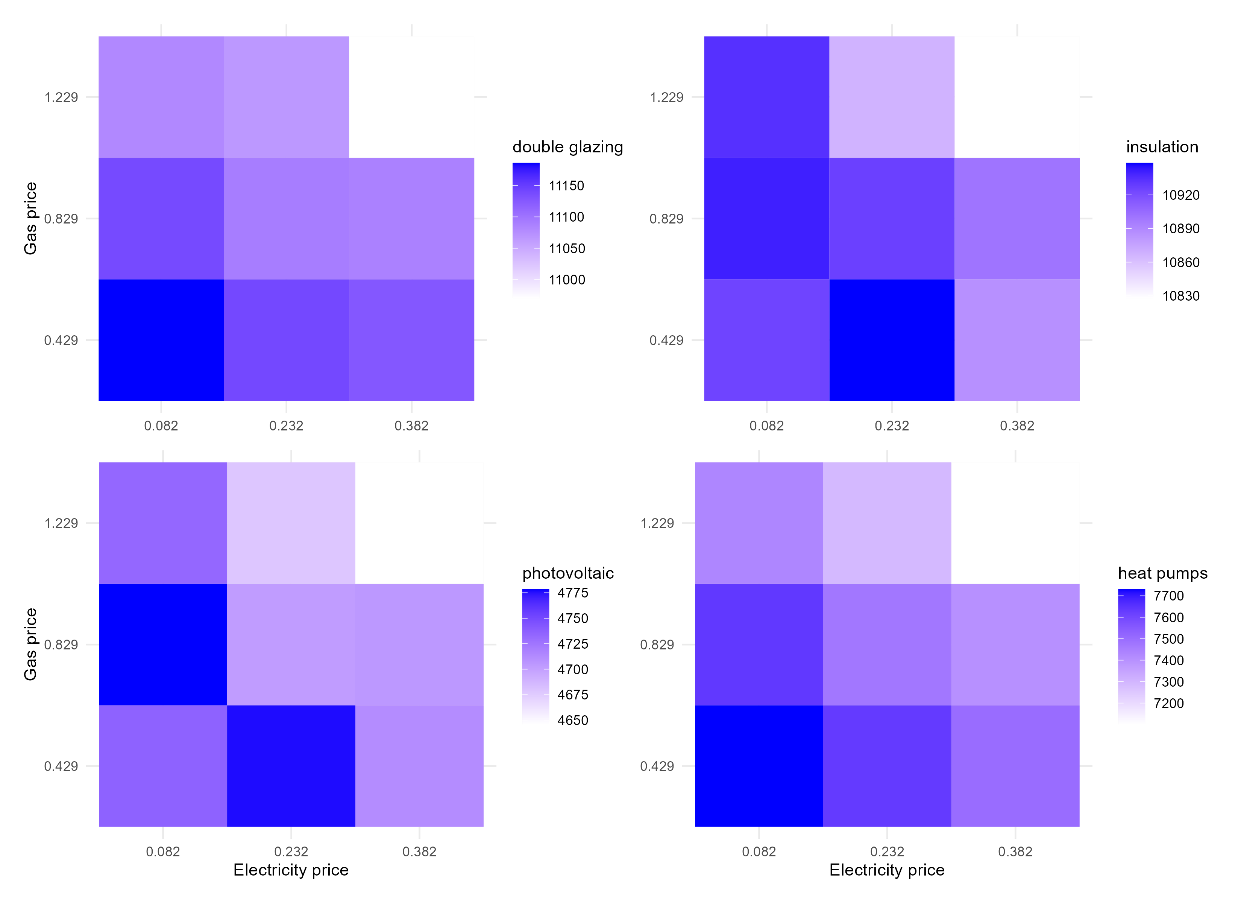


Figure C. 9. The effect of the interaction of gas and electricity price changes on the EER uptake in Noord


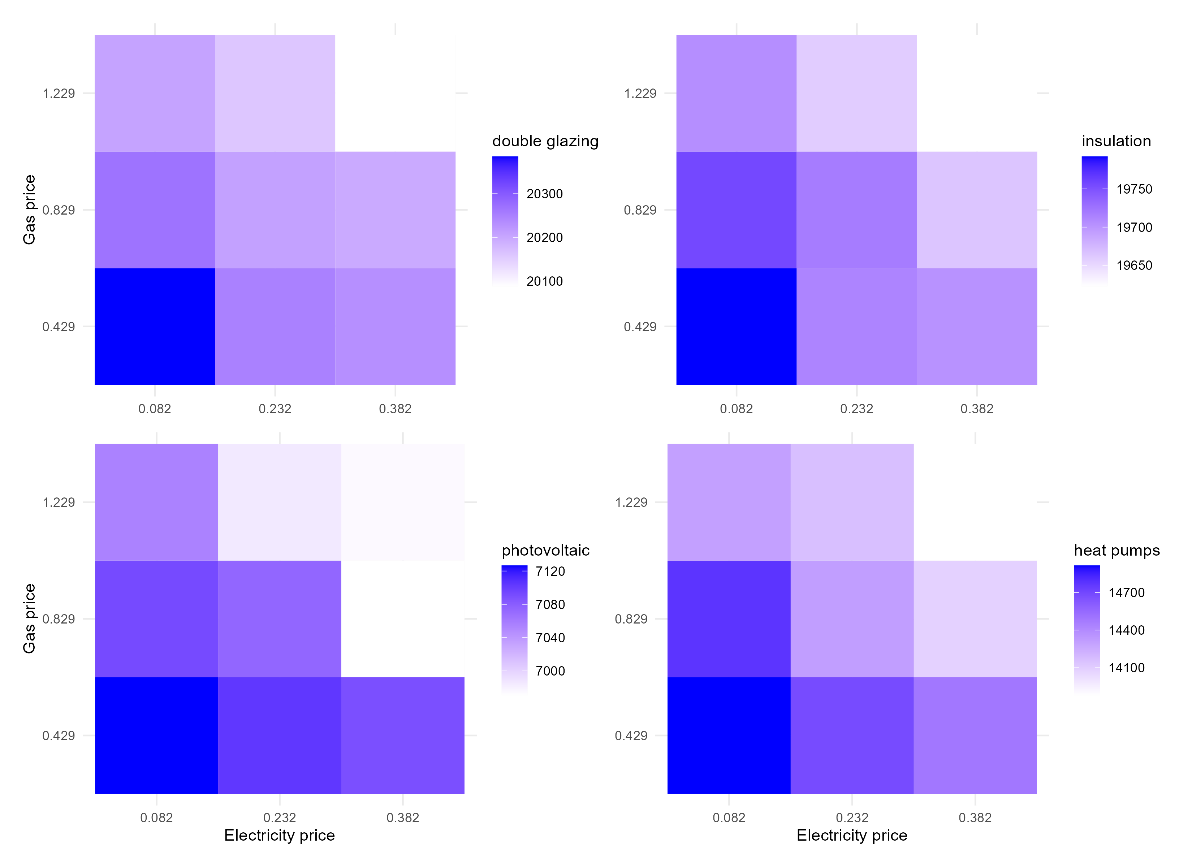


Figure C. 10. The effect of the interaction of gas and electricity price changes on the EER uptake in West
